# Summer warming and deoxygenation shape estuarine microbial plankton across domains of life

**DOI:** 10.1093/ismeco/ycag015

**Published:** 2026-01-22

**Authors:** Jodi Dharam, Pukhraj Kaur, Amedea Cipriano, Abigail Salgado, Aleena Qureshi, Khabiba Shahid, Carissa Kissoon, Fabian Leija, Luciana Santoferrara

**Affiliations:** Department of Biology, Hofstra University, Hempstead, NY, United States; Department of Biology, Hofstra University, Hempstead, NY, United States; Department of Biology, Hofstra University, Hempstead, NY, United States; Department of Biology, Hofstra University, Hempstead, NY, United States; Department of Biology, Hofstra University, Hempstead, NY, United States; Department of Biology, Hofstra University, Hempstead, NY, United States; Department of Biology, Hofstra University, Hempstead, NY, United States; Department of Biology, Hofstra University, Hempstead, NY, United States; School of Marine and Atmospheric Sciences, Stony Brook University, Stony Brook, NY, United States; Department of Biology, Hofstra University, Hempstead, NY, United States

**Keywords:** hypoxia, eutrophication, bacteria, archaea, microbial eukaryotes, protists, biomass, diversity

## Abstract

Microbial plankton have complex relationships with interacting coastal changes, including warming, eutrophication, and deoxygenation. At intra-annual scales, these changes can be tracked during the summer progression of warming and deoxygenation in eutrophic, seasonally hypoxic coasts. To study these dynamics, we combined flow cytometry (prokaryote biomass), microscopy (biomass of consumer protists and micrometazoans), and DNA metabarcoding (diversity of prokaryotes and microbial eukaryotes) in a harbour of the Long Island Sound estuary (New York, USA) over two summers. We found that biomass values remained relatively stable, whereas alpha- and beta-diversity differed significantly between pre-hypoxic and hypoxic periods. Among more than 10 abiotic and biotic variables analysed, temperature emerged as the most significant predictor of prokaryote biomass, and both prokaryote and microeukaryote alpha- and beta-diversity. Yet shifts in taxonomic profiles, with higher proportions of prokaryote N- and S-cyclers and protistan parasites during hypoxia, suggest functional changes more strongly linked to lower dissolved oxygen (DO) than to higher temperatures. Overall, we revealed that summer warming has a stronger influence on microbial biomass and diversity than deoxygenation, while the latter covaries with taxa that have specific biogeochemical roles (e.g. ammonia oxidation with consequent DO consumption) or biological interactions (e.g. parasitism). These findings underscore the importance of temperature and DO in structuring microbial plankton across domains of life and provide insight into potential microbial responses (or contributions) to environmental stress.

## Introduction

Marine and estuarine coastal environments are experiencing multifaceted changes driven by global and local anthropogenic pressures. Among the most pressing and interconnected changes are warming, eutrophication, and deoxygenation, each of them potentially altering the structure, function and ecosystem services of microbial communities [1, 2]. Warming accelerates metabolic rates and affects chemical fluxes by decreasing gas solubility and favouring water-column stratification [3, 4], while nutrient enrichment from agricultural runoff and urban wastewater boosts primary production and microbial oxidative reactions [5]. Concurrently, these processes exacerbate deoxygenation in bottom waters and underlying sediments, which alters microbial metabolisms and associated biogeochemical cycles, and can enhance the production of greenhouse gases thus causing positive feedbacks on warming [6, 7].

Microbial plankton span the three domains of life (Bacteria, Archaea, Eukarya) and have intricate relationships with natural and anthropogenic changes in coastal systems. Some members of the microbial community cause environmental fluctuations, e.g. via eutrophication-fuelled microalgal blooms and prokaryotic oxidation of the produced organic matter at the expense of dissolved oxygen (DO) [8]. While the consequent deoxygenation favours prokaryotes with microaerobic and anaerobic metabolisms [9], it can negatively impact obligate aerobes, as exemplified by reduced growth rates in protists (e.g. ciliates, dinoflagellates [10, 11]), and micrometazoans (e.g. copepod nauplii [12]). Taxon-specific thermal optima further influence growth and metabolism across microbial plankton [13–15] and may compound or counteract the effects of deoxygenation [11, 16]. Superimposed to abiotic drivers is a vast network of microbial activities and interactions, including various organo- and lithotrophic prokaryote metabolisms, primary production by phytoplankton, grazing by protozooplankton and micrometazoans, combination of photo- and phago-trophy in mixoplankton, endosymbiotic and parasitic relationships, and viral lysis, all of which strongly influence carbon flux and nutrient recycling and are susceptible to change [17, 18].

Microorganisms have fundamental roles in food webs, biogeochemical cycles, and climate regulation, and thus their influences on and responses to coastal environmental changes urgently need clarification, although the robust quantification of these feedbacks is precluded by major drawbacks and knowledge gaps [19]. Crucial mechanistic insights derive from experimental studies that are too laborious to focus on multiple factors or on more than a few species [20], and observational analyses in the context of interactive changes have rarely considered the full spectrum of microbial plankton, even for well-known linked phenomena like coastal eutrophication and hypoxia [21]. Furthermore, anthropogenic changes are superimposed to the natural variations experienced by microbial communities, e.g. seasonal temperature and primary production changes in temperate ecosystems [22, 23] or redox potential fluctuations across natural oxygen-deficient zones [24]. Yet, the long-term data needed for a reliable understanding on microbial and environmental feedbacks is limited [25]. Meanwhile, the study of intra-annual dynamics provides a valuable baseline and, cautiously taken, offers insight on *in situ* restructuring of microbial communities in correlation to key environmental drivers (e.g. [26]).

Here we analysed microbial community changes in a eutrophic, seasonally hypoxic harbour during the progression of warming and deoxygenation over two summer seasons. Our goal was to quantify the influence of temperature, DO and other abiotic and biotic factors on microbial biomass and diversity changes across domains of life. Key questions included: Does the community structure of microbial plankton change significantly between normoxic and hypoxic periods? Is deoxygenation significantly related to variations in microbial biomass and diversity? Are taxon-specific temporal distributions linked to environmental variations over the summer? To address these questions, we combined flow cytometry (prokaryote biomass), microscopy (biomass of consumer protists and micrometazoans), DNA metabarcoding (diversity of prokaryotes and microbial eukaryotes), and statistical approaches aimed at modelling multiple explanatory variables in order to identify key environmental drivers of microbial plankton communities.

## Material and methods

### Sampling, environmental variables, and microbial biomass

A detailed version of this section is provided in the Supplementary Methods. In brief, this study was conducted within Long Island Sound (LIS), a temperate estuary that borders with New York City and its suburbs. Specifically, we studied Hampstead Harbor (Fig. S1), a 5-miles long harbour that presents bottom-water hypoxia (DO <3.0 mg L^−1^) every summer [27]. We sampled four inland-to-open-estuary stations (Fig. S1) at surface and bottom depths (0.5 m below the surface and 0.5 m above the bottom, respectively) from a boat over 10 dates in the summers of 2022 and 2023 (68 samples in total; Table S1). Water temperature, salinity, DO, pH, chlorophyll *a* concentration, and turbidity were recorded with a multiparameter reader (Manta+35, Eureka Water Probes, Texas in 2022; and EXO2S, YSI, Ohio in 2023). Water samples were collected with a Van Dorn bottle and immediately stored for nutrient quantifications or preserved for microscopy (2% non-acid Lugol’s solution). Water aliquots gently passed through a sieve (250-μm pore size) were preserved for flow cytometry (1% paraformaldehyde plus 0.05% glutaraldehyde; only in 2023) and filtered (500 ml) through 0.2-μm-pore polycarbonate membranes (MilliporeSigma, Massachusetts) for DNA extraction.

Nitrite, nitrate, ammonia, and total Kjeldahl (ammonia + organic) nitrogen concentrations were determined by colorimetry (Pace Analytical, New York) and used to calculate total inorganic and organic nitrogen (TIN and TON, respectively). Prokaryote abundance (only in 2023) was estimated using a FACSVerse flow cytometer (Becton, Dickinson and Company, New Jersey) and published protocols [28], and then converted into biomass [29]. Within microeukaryotes, we estimated the abundance of consumer organisms, or microconsumers, by inverted microscopy [30]. We focused on primarily consumers (i.e. depending on grazing): protozooplankton and non-constitutive mixoplankton (ciliates, dinoflagellates [31, 32]) as well as micrometazoans (copepod nauplii and rotifers) in the ~20–200 μm size fraction. Primarily photosynthesizers (phytoplankton and constitutive mixoplankton) were not quantified. At least 200 (median = 623) specimens per sample were counted using a DMi8 inverted microscope (Leica, Illinois) and classified based on shape and size. Random specimens were measured with the LAS X software (Leica, Illinois) for biovolume calculations, then used for biomass estimates based on group-specific conversion factors (Supplementary Methods).

### DNA extraction, sequencing, and raw-sequence processing

A detailed version of this section is provided in the Supplementary Methods. In brief, DNA was extracted with the Fecal/Soil Microbiome kit D6012 (Zymo Research, California) and used to separately amplify the V4 regions of the 16S and 18S rRNA genes (abbreviated as 16S or 18S from now on) with primers for prokaryotes [33, 34] and microeukaryotes [35], respectively. Triplicate PCR products per sample were pooled for cleaning, quality assessment, and MiSeq paired-end sequencing (Illumina Inc., California) at the Microbial Analysis, Resources and Services facility, University of Connecticut. As expected, simultaneous sequencing of negative controls yielded a negligible number of reads, while the ZymoBIOMICS Microbial Community DNA Standard D6305 (Zymo Research, California) produced an even distribution of amplicon relative abundances. A total of 11.5 million 16S and 18S reads were generated (N = 66 and 68 samples, respectively, as two samples failed for 16S) and the raw datasets are available in NCBI’s Sequence Read Archive (accession number PRJNA1329165).

DNA sequences were demultiplexed, quality-filtered, and dereplicated into amplicon sequence variants (ASVs) using the DADA2 plugin [36] in QIIME 2 [37]. Taxonomic classification of ASVs used a Naive Bayes classifier trained on V4-trimmed versions of SILVA v. 138 for prokaryotes [38] and PR2 v. 5.0.0 for microeukaryotes [39, 40]. ASVs annotated as chloroplast, mitochondrion, or non-target sequences were eliminated from both datasets.

### Data analysis

Diversity estimates and multivariate ordinations based on DNA were done in QIIME2 [37] and in R v. 3.6.3 [41] using the *vegan* package [42]. To prepare data for the analyses, the prokaryote and microeukaryote DNA datasets were subsampled to the minimum number of total reads per sample (11 485 and 8785 reads for 16S and 18S, respectively) and environmental metadata were standardized to scale each variable to a zero mean and unit variance. Alpha-diversity was computed using the Shannon index. To evaluate the completeness of our sampling and the effect of subsampling on alpha-diversity, we repeated estimates at increasing subsampling steps of 1000 reads at the time, with 100 iterations per step. Beta-diversity was computed with principal coordinates analyses (PCoA) based on Bray–Curtis dissimilarity matrices. To evaluate the effect of subsampling on beta-diversity, each DNA dataset subsampling was iterated 1000 times to calculate jack-knifed PCoAs and Spearman’s correlations between pairwise iterations. We also conducted ordinations based on Aitchison distance matrices (Euclidean distances based on centred log-ratio transformed non-subsampled reads and added pseudo-count of 1 for zeros), which are expected to minimize the effect of uneven total reads per sample and compositional bias inherent in metabarcoding data [43]. The Bray-Curtis and Aitchinson PCoA results were similar, but the later were possibly affected by the unequal number of total reads obtained in 2022 and 2023, a bias better handled by subsampling [44] and thus this is our retained approach. Beta-diversity differences between periods were evaluated with the Analysis of Similarities (ANOSIM) test using the Bray-Curtis matrices and 999 permutations [45]. The correlation between Bray–Curtis dissimilarity (sequence) and Euclidean distance (environmental) matrices was tested with Mantel tests using 999 permutations [46]. The subsets of environmental variables that best correlated with the Bray-Curtis matrices were then identified with the BIOENV test [47]. Association networks among bacteria, archaea and microeukaryotes were built with SpiecEasi [48] as implemented in the NetCoMi R package v.1.2 [49] (Supplementary Methods).

Correlations among environmental variables were calculated using Pearson’s coefficient, while correlations between environmental variables and taxa reads were based on Spearman’s coefficient. Differences among periods in environmental variables, biomass, and Shannon index were tested using the Kruskal–Wallis test. When significant differences were found, pairwise comparisons were conducted using Dunn’s test for pairwise comparisons. All the correlation and comparison analyses used the Benjamini–Hochberg adjustment for multiple testing.

General Additive Models (GAMs) were used to predict the best explanatory variables for biomass and Shannon diversity index values. GAMs were built with the mgcv R package, using the restricted maximum likelihood method for smoothing parameter estimation and automatic optimization of effective degrees of freedom (EDF) [50, 51]. GAMs were run separately for prokaryotes and microeukaryotes, iteratively considering different combinations of abiotic and biotic variables (temperature, salinity, DO, pH, chlorophyll *a*, turbidity; to evaluate the effect of potential interactions, prokaryote biomass and Shannon index were also included in microeukaryote models and vice versa). Models for which variables yielded high concurvity (autocorrelation among variables; threshold = 0.8) and/or no explanatory power (p-value >0.05 for all variables) were discarded. EDF values were used to indicate the complexity of the relationship between explanatory and response variables, with EDF = 1 equalling a linear relationship and higher EDF values meaning increasingly non-linear relationships. Final models were assessed for normality and homogeneity of variance using standardized residual plots and Q-Q plots [50].

## Results and discussion

### Environmental conditions

Contrasting climate conditions were reported for the 2022 and 2023 summers. The area experienced abnormally dry conditions in summer 2022, while record rain was observed in summer 2023 [27]. Accordingly, average cumulative precipitation during the week before each of our samplings was 1.6 times lower in 2022 (13.5 mm) than in 2023 (21.5 mm), and average salinity values were higher in 2022 (26.7) than in 2023 (25.9; Table S1). Both summers presented pre-hypoxic and hypoxic periods (see below), with hypoxia lasting ~35 days each year and being associated with fishkills in 2022 but not in 2023 [27].

**Figure 1:**
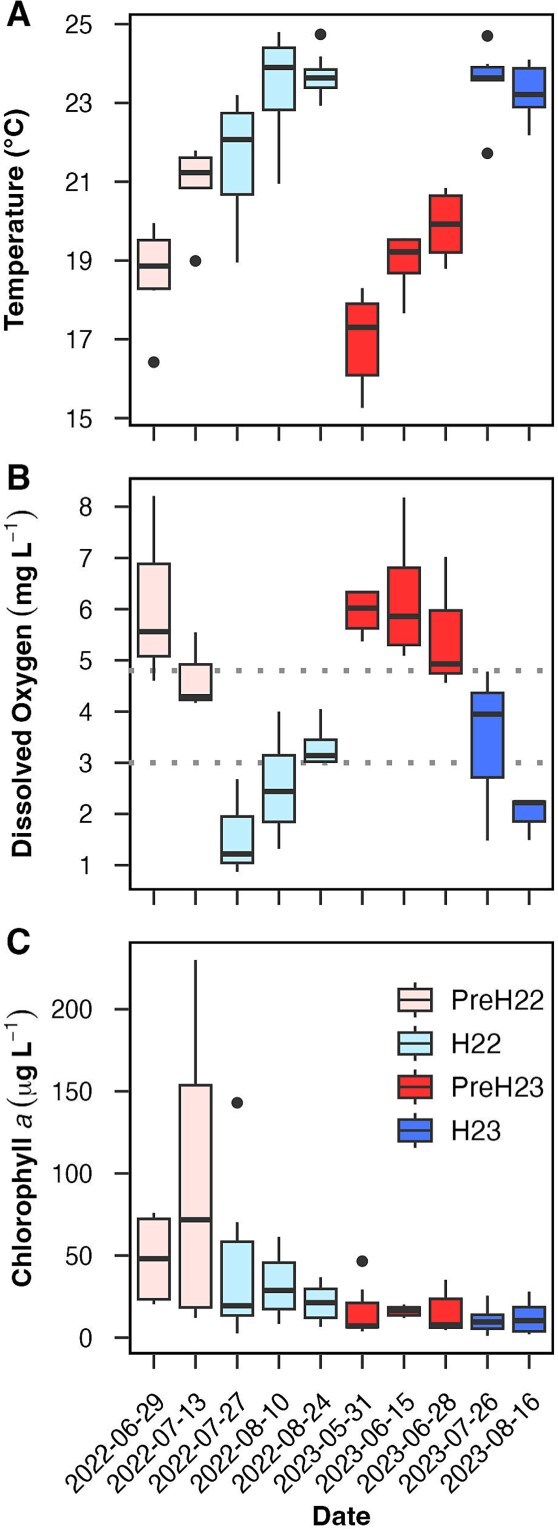
Key environmental variables (see Fig. S2 for other variables). (A) water temperature, (B) bottom-water dissolved oxygen (DO) and (C) chlorophyll *a* concentration per date during pre-hypoxia (PreH) and hypoxia (H) in 2022 and 2023 (N = 68 in A and C including both surface and bottom waters; N = 34 in B). Boxplots show medians (thick line), interquartile ranges (IQR, boxes), ranges within 1.5 times the IQR (whiskers) and outliers. In B, dotted lines indicate hypoxic and suboptimal DO thresholds [27]. Kruskal-Wallis tests among periods were significant (p-value <0.01) for all three variables; Dunn’s comparisons were significant (p-value <0.01) for temperature and DO between pre-hypoxic and hypoxic periods within each year, and for chlorophyll *a* in pre-hypoxia 2022 versus all other periods.

Despite the differences in climate and sampling timeframes in 2022 (late June to late August) and 2023 (late May to mid-August), we recorded similar ranges and trends in water temperature and DO during both summers (Fig. 1A and B). Median temperatures (surface and bottom) increased significantly (by 5–6°C) throughout each summer, with only a slight decrease (~0.3°C) during the last sampling date of each year. Bottom-water DO decreased significantly throughout both summers, with median values halving from pre-hypoxia (5.1–5.7 mg L^−1^) to hypoxia (2.2–2.9 mg L^−1^). During both hypoxic periods, 69% of the bottom samples had DO levels below 3.0 mg L^−1^ (minimum = 0.9 mg L^−1^), and the rest had suboptimal DO levels (3.0–4.8 mg L^−1^) as defined for the area [27]. Temperature and DO had significant correlations between themselves and with sampling depth, salinity, and pH (Table S2): As sample depth increased, temperature, DO, and pH decreased, while salinity raised. The inverse (but weak) relationship between DO and temperature is expected based on oxygen solubility, while DO and pH had the strongest correlation among environmental variables, consistent with reports that deoxygenation and acidification are directly linked in LIS [52].

Chlorophyll *a* concentration was significantly higher during the pre-hypoxic period in 2022, with two to 10 times increased median values than during all other periods (Fig. 1C). Maxima in chlorophyll *a* (117–230 μg L^−1^) and ammonia (0.3 mg L^−1^) recorded in pre-hypoxia 2022 (station 1, July 13) up to six times higher than recorded in the main stem of LIS [21, 53] indicate that the harbour can reach higher eutrophication levels than the adjacent open estuary. Chlorophyll *a* was significantly and negatively correlated with sample depth but was not statistically linked to other environmental variables we quantified (Table S2). Nitrite, nitrate, ammonia, TIN, and TON concentrations were generally below the detection limits of our method (Table S1), with maxima up to 0.3 mg L^−1^ for TIN and 3.4 mg L^−1^ for TON in both pre-hypoxic periods examined.

### Biomass of prokaryotes and microconsumers

**Figure 2:**
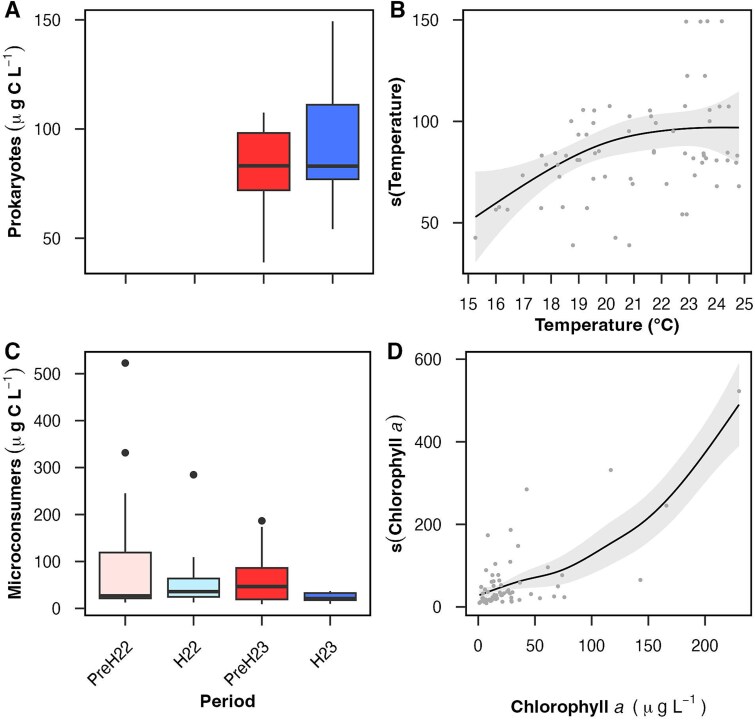
Biomass. (A) prokaryote and (C) microconsumer biomass values during pre-hypoxia (PreH) and hypoxia (H) in 2022 and 2023 (including both surface and bottom waters; N = 34 in A as not data is available for 2022; N = 64 in C). Boxplots as detailed in Fig. 1. Kruskal–Wallis tests among periods were not significant (p-value >0.05). Abundance and biomass values per group and date are shown in Fig. S3 and S4. Generalized additive model (GAM) results for prokaryote (B) and microconsumer (D) biomass. A given smooth function, or “s(explanatory variable),” represents the predicted biomass as a function of that explanatory variable; only significant smooth functions are included. The shadowed area indicates the 95% confidence interval. Additional GAM diagnostics are provided in Table 1.

Biomass did not change significantly between pre-hypoxic and hypoxic periods (Fig. 2A and C) or between years, dates, stations or depth layers (not shown). Prokaryote biomass had median values of 83 μg C L^−1^ during both pre-hypoxia and hypoxia 2023. Prokaryote maxima (7.5 × 10^6^ cells ml ^−1^ and 149 μg C L^−1^; Figs S3A and S4A) were in the same order of magnitude than the summer maxima found by flow cytometry in other temperate estuaries [54–57]. Microconsumer biomass was more variable across the four periods we investigated, with median values ranging from 21 to 47 μg C L^−1^ (Table S3). Outlier values of 332 and 523 μg C L^−1^ during pre-hypoxia 2022 were due to peaks in the biomass of microconsumer dinoflagellates recorded in station 1 (bottom and surface, respectively) during July 13 (Fig. S4). Aloricate and loricate ciliates had maximum values of 125 and 24 μg C L^−1^, respectively, while copepod nauplii and rotifers only contributed a maximum of 7–10 μg C L^−1^ each (Fig. S4). Like prokaryotes, maxima for each microconsumer group (Figs S3 and S4) were comparable to those reported in other temperate estuaries during the summer [58–60].

Generalized Additive Models (GAMs) indicated non-linear relationships between microbial biomass and environmental variables (Fig. 2B and D; Table 1). The GAM with the highest explanatory power for prokaryote biomass showed a significant, positive relationship with temperature that became asymptotic at ~22°C, and a non-significant relationship with chlorophyll *a*. The GAM with the highest explanatory power for microconsumer biomass included temperature, DO, and chlorophyll *a*, although only the latter yielded a significant, positive trend. The explained deviance was higher for microconsumers (61%) than for prokaryotes (34%) and it was not significantly increased by other abiotic and biotic factors we measured.

**Table 1:** Generalized additive model (GAM) results for microbial biomass. N = number of samples; R^2^ = model’s coefficient of determination; EDF = effective degrees of freedom (trends based on values closer to 1 = linear, 2 = quadratic, 3 = cubic); F statistic and p-value refer to the significance of smooth functions. Significant results are shown in bold.

Group	N	R^2^	Deviance	Smooth function	EDF	F	p-value
Prokaryotes	34	0.27	34%	**s(Temperature)**	1.75	4.47	**0.02**
				s(Chlorophyll *a*)	1.63	1.46	0.25
Microconsumers	64	0.58	61%	s(Temperature)	1.46	0.25	0.77
				s(Dissolved Oxygen)	1.00	0.05	0.83
				**s(Chlorophyll *a*)**	2.95	22.55	**<2e** ^**−16**^

The fundamental role of temperature on the regulation of microbial growth and metabolism is well established [61]. Our finding of a significant, positive relationship between prokaryote biomass and temperature in a range of 15–22°C (Fig. 2B, Table 1) is consistent with previous reports in temperate estuaries [55, 62]. Interestingly, there is a match in the temperature (~22°C) corresponding to both our maximum prokaryote biomass (Fig. 2B) and the maximum bacterioplankton production found in various temperate estuaries [63]. Once production peaks and growth efficiency drastically decreases at levels higher than 22°C [63], it is likely that biomass accumulation stops due to physiological stress of at least some taxa [64] or increased top-down regulation. Viral lysis and/or bacterivory were likely more relevant than bottom-up controls (quantity and quality of dissolved organic carbon and inorganic nutrients) in the system we analysed, as suggested by chlorophyll *a* and N-compound concentrations not emerging as relevant drivers of prokaryote biomass in our analyses (Table 1) or other eutrophic systems [65–67].

The strong, positive relationship between microconsumer biomass and chlorophyll *a* (Fig. 2D, Table 1) is an expected result usually attributed to co-occurrence with chlorophyll-containing prey ([68] and references therein). Here, this relationship was partly driven by maxima recorded simultaneously for microconsumer biomass and chlorophyll *a* during pre-hypoxia 2022 (station 1, July 13), when dinoflagellates with different trophic capabilities contributed to each variable: protozooplanktonic *Polykrikos* and *Gyrodinium* spp. and chlorophyll-containing (auto- or mixoplanktonic) *Prorocentrum* spp. (Fig. S5). The later were likely stimulated by some of the maxima ammonia and TON concentrations recorded during our study (up to 0.3 and 1.0 mg L^−1^; Table S1), consistent with organic nitrogen favouring dinoflagellate- over diatom-dominated phytoplankton in LIS [53].

### Alpha-diversity of prokaryotes and microeukaryotes

**Figure 3:**
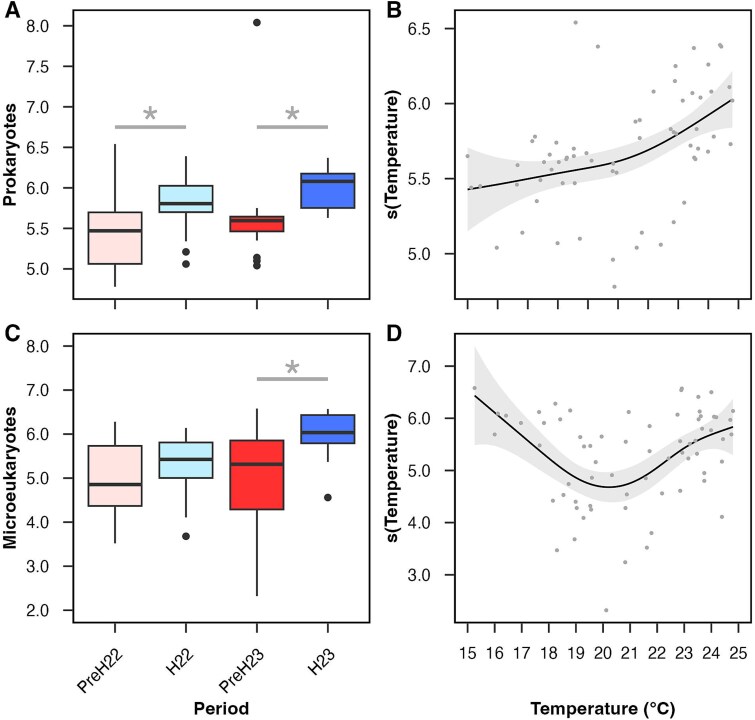
Alpha-diversity based on the Shannon index. (A) prokaryote and (C) microeukaryote values during pre-hypoxia (PreH) and hypoxia (H) in 2022 and 2023 (including both surface and bottom waters; N = 66 in A; N = 68 in C). Boxplots as detailed in Fig. 1. Kruskal–Wallis tests among periods were significant (p-value <0.01); asterisks indicate Dunn’s test significance (p-value <0.01) for comparisons within each year. Generalized additive model (GAM) results for prokaryote (B) and microeukaryote (D) Shannon index. GAMs as detailed in Fig. 2; only the most significant smooth functions are included (less significant ones are shown in Fig. S7). Additional GAM diagnostics are provided in Table 2.

Shannon index values saturated well below the subsampling levels we used for either prokaryotes or microeukaryotes, suggesting that our study captured most of the diversity during each date and period analysed (Fig. S6). Values changed significantly between pre-hypoxic and hypoxic periods (Fig 3A and C) but not between years, dates (with few exceptions), stations or depth layers (not shown). Shannon index median values were 6%–14% higher in hypoxia than in pre-hypoxia (Table S3) and these increases were significant for prokaryotes in both years analysed, and for microeukaryotes in 2023 (Fig 3A and C).

GAMs identified temperature as the most significant driver of changes in prokaryote and microeukaryote Shannon index values (Fig 3B and D; Table 2). For prokaryotes, the relationship with temperature was positive (Fig. 3B), and the model also included less significant effects of DO and chlorophyll *a* (Fig. S7). For microeukaryotes, the trend with temperature was only positive between 21–25°C, and the model also included a non-significant relationship with chlorophyll *a*. The explained deviance was similar for the prokaryote and microeukaryote GAMs (44%–46%) and it was not significantly increased by other abiotic and biotic factors we measured. Using an alternative alpha-diversity metrics (Faith’s phylogenetic diversity index) yielded similar trends compared to the Shannon index for both prokaryotes and microeukaryotes (Fig. S8).

**Table 2:** Generalized additive model (GAM) results for the Shannon index. Details as in Table 1.

Group	N	R^2^	Deviance	Smooth function	EDF	F	p-value
Prokaryotes	66	0.40	46%	**s(Temperature)**	1.80	6.04	**<4e** ^**−3**^
				**s(Dissolved Oxygen)**	1.43	3.17	**0.04**
				**s(Chlorophyll *a*)**	3.08	3.44	**0.01**
Microeukaryotes	68	0.36	44%	**s(Temperature)**	3.26	6.88	**<2e** ^**−4**^
				s(Chlorophyll *a*)	4.87	1.79	0.10

Our finding of temperature as the main driver of changes in alpha diversity matches global trends for marine prokaryotic and eukaryotic plankton [69]. A positive relationship between microbial alpha-diversity and temperature is best known based on global latitudinal changes and it has been attributed to warmer temperatures accelerating metabolic rates, in turn increasing growth and mutation rates, and ultimately stimulating speciation and higher diversity [69, 70]. This paradigm, however, does not always adjust to observations at smaller spatial scales and higher temporal resolutions. For example, temperature frequently has an influence on, but is not always the main driver of, the variability in the Shannon index or other measurements of alpha-diversity in other temperate estuaries and coasts over time, or sometimes this relationship is negative [71–75]. Thus, local or regional abiotic and biotic factors likely add complexity to the alpha-diversity and temperature covariation, and tendencies may also be affected by methodological differences. For example, the negative trend we observed for microeukaryote alpha-diversity below 20°C may be due to overlaps in Shannon index and temperature minima in May, which was only sampled in 2023 (Fig 1A and 3C); a higher temporal resolution (e.g. weekly over multiple years) could help us fully elucidate this relationship.

Another factor often interrogated in relation to marine microbial alpha-diversity is primary production (usually with chlorophyll *a* as a proxy), under the assumption that greater resource availability promotes higher diversity via niche diversification and species coexistence, although support has rarely been found [69, 76, 77]. Similarly, we only found a marginally significant relationship between chlorophyll *a* and prokaryote Shannon index (Fig. S7A; Table 2). Finally, although less significant, the negative trend between prokaryote Shannon index and DO (Fig. S7B) matched findings in other seasonally hypoxic estuaries [78, 79] and in a preliminary study we conducted in adjacent, open-estuary LIS waters with a much narrower temporal resolution of only two dates [21]. This increase in prokaryote alpha-diversity with deoxygenation may be due to habitat heterogeneity across DO gradients, where contiguous niches for taxa with distinct oxygen requirements overlap [21, 80]. Given the GAM results for prokaryotes and microeukaryotes (Fig 3B and D), DO alone does not explain the significant differences in alpha-diversity observed between pre-hypoxic and hypoxic periods (Fig 3A and C), as further discussed below.

### Beta-diversity of prokaryotes and microeukaryotes

**Figure 4:**
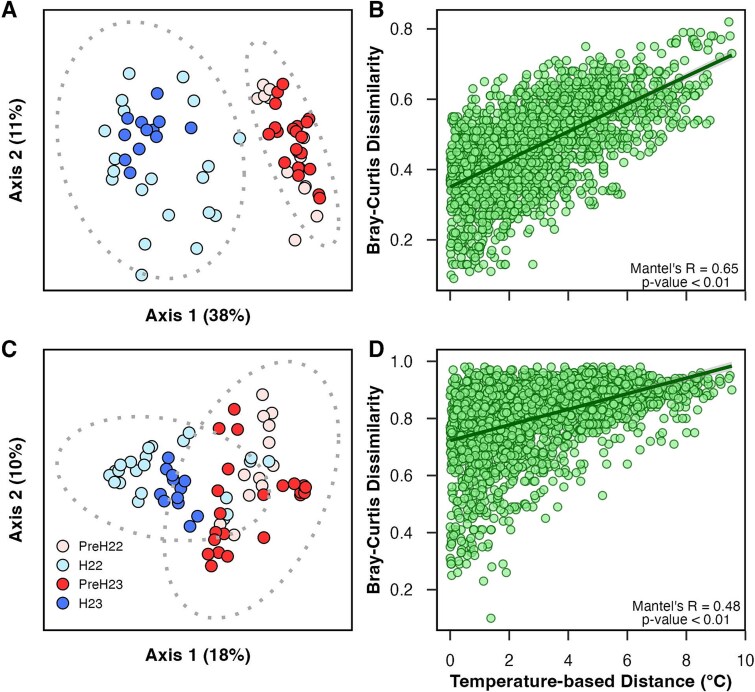
Beta-diversity. (A) prokaryote and (C) microeukaryote principal coordinates analyses (PCoA) based on bray–Curtis dissimilarity matrices (including both surface and bottom waters; N = 66 in A; N = 68 in C). Samples from pre-hypoxia (preH) and hypoxia (H) in both 2022 and 2023 (ellipses) were significantly different based on ANOSIM (R = 0.78 for prokaryotes and 0.48 for microeukaryotes; p-value <0.01). Jack-knifed PCoAs (not shown) presented the same sample distributions and Spearman’s coefficients between pairwise iterations were always >0.99, indicating no effect of subsampling on beta-diversity estimates. PCoAs coloured by date or based on Aitchison distance matrices are show in Figs S9 and S10, respectively. Bray–Curtis dissimilarity versus temperature-based distance for prokaryotes (B) and microeukaryotes (D) showing mantel test results, linear fit (dark green line) and 95% confidence interval (grey).

PCoA and ANOSIM analyses based on Bray–Curtis dissimilarity indicated significant changes in prokaryote and microeukaryote beta-diversity between pre-hypoxic and hypoxic periods (Fig 4A and C). Overlapping, more gradual changes in beta-diversity were observed from the first to the last date of sampling in both 2022 and 2023 (Fig. S9). These results indicate that sampling period and date (but not year, station or depth layer) drove the observed changes in the microbial community structure. The temporal gradient observed within each summer (Fig. S9) indicates that prokaryotic and microeukaryotic communities were more similar between years than across each set of five dates studied in 2022 and 2023. Yearly recurrence has also been observed for bacterioplankton and/or microbial eukaryotes in other temperate coasts over annual time series spanning up to a decade, with summer often being among the most recurrent times of the year [74, 81–83]. This confirms the impact of intra-annual temporal variations on microbial community structure, which can override the effects of spatial heterogeneity across sites or depths at local scales affected by seasonality [73].

Temperature emerged as the most relevant variable explaining beta-diversity out of all possible combinations of environmental factors we measured (BIOENV test; Table S4). Furthermore, positive significant correlations between temperature and Bray-Curtis dissimilarities were observed for both prokaryotes and microeukaryotes (Fig. 4B and D). In other words, the more different samples were in temperature, the more contrasting their microbial communities were. We found these community differentiations even if our observed gradient of ~10°C (Fig 4B and D) is a fraction of the temperature variations found to drive the beta-diversity of bacterioplankton over ~20°C through full seasonal cycles in temperate coasts [81, 84] and of bacterioplankton and picoeukaryotes across ~30°C from subarctic to subantarctic waters [85]. This supports the key influence of temperature on structuring microbial communities across magnitudes of temperature change and in both the temporal and spatial axes. The question remains whether this pivotal role of temperature is due to differences among species (or at other levels behind beta-diversity, such as ASVs here) in thermal optima for growth [86–88], ability to use resources that are differentially available across temperature gradients [89], and/or temperature-specific biological interactions [90].

DO played a marginal role in explaining prokaryote and microeukaryote beta-diversity, as it only appeared in the third rank of the BIOENV results in combination with temperature and salinity (Table S4). Thus, like for alpha-diversity, community differentiations in the pre-hypoxic and hypoxic periods (Fig 4A and C) were likely not caused by DO per se, but instead by a combination of factors that changed over the summer, in agreement with our preliminary findings in adjacent LIS waters [21]. The hypoxia intensity (reaching DO levels as low as 0.9 mg L^−1^) and persistence (lasting 35–45 days) in this system during our surveys (Table S1; [21, 27]) did not result in DO being the main driver of changes in prokaryote or microeukaryote biomass, alpha- or beta-diversity (Tables 1, 2, Table S4). Similarly, other studies reporting changes in prokaryote or microeukaryote community structure during seasonal or episodic hypoxia in other temperate coastal environments have not shown DO as the sole or main explanatory factor [9, 79, 80, 91]. We thus hypothesize that, at least for prokaryotes, deoxygenation may have a less direct effect on community structure than on functioning, given that many aerobic species can survive low oxygen levels or facultatively switch to anaerobic metabolisms [9]. However, switches from aerobic to anaerobic metabolisms detected by metatranscriptomics during summer deoxygenation in another temperate estuary were, again, better explained by factors (depth) other than DO [92].

### DNA-based taxonomic composition and potential functional shifts during hypoxia

The prokaryote and microeukaryote communities were dominated by taxa that also presented widespread distributions (Figs S11 and S12). For prokaryotes, 71% of the reads corresponded to taxa prevailing in most samples, including Alphaproteobacteria (SAR11 Ia, SAR116, Rhodobacterales), Gammaproteobacteria (SAR86, Cellvibrionales, Oceanospirillales), Bacteroidota (Flavobacteriales), and Actinobacteriota (Actinomarinales). For microeukaryotes, the widespread and prevalent taxa (49% of reads) included protists in the TSAR supergroup (Dinoflagellata, Diatomeae, Cercozoa), Chlorophyta and Cryptophyta, as well as copepods (Maxillopoda). These taxa are ubiquitous in marine environments and among the most abundant groups in temperate estuaries that have been studied for prokaryotes [57, 75, 78, 79, 93] or including also microeukaryotes [21, 26]. Thus, the bulk of the microbial community at broad taxonomic ranks seems well adapted to estuarine variability.

**Figure 5:**
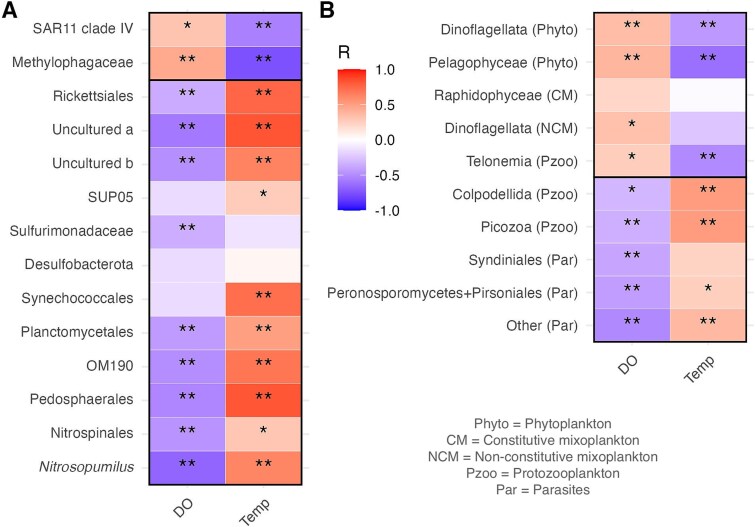
Taxa differentially distributed between the pre-hypoxic and hypoxic periods and their correlations with dissolved oxygen (DO) and temperature (temp). (A) prokaryotes (N = 66) and (B) microeukaryotes (N = 68). Spearman’s coefficients (R) and significance after applying the Benjamini-Hochberg adjustment for multiple testing (*p-value <0.05; **p-value <0.01) are shown. Full taxa names and distributions are shown in Figs S11 and S12. Only the least inclusive taxonomic ranks (and trophic groups for microeukaryotes) are shown here.

Taxa differentially distributed during the pre-hypoxic and hypoxic periods (Figs S11 and S12) were significantly correlated with DO, temperature or both (Fig. 5). Pre-hypoxia bacteria (SAR11 IV Alphaproteobacteria, Methylophagaceae Gammaproteobacteria) and protists (Pelagophyceae, Raphidophyceae, Telonemia) generally had significant links to higher DO and lower temperatures. Among Dinoflagellata, phytoplanktonic and parasitic taxa had widespread distributions, but were more prevalent under cooler normoxic waters or at lower DO levels, respectively. Mixoplanktonic taxa were weakly but positively correlated with DO. Protists found mostly during hypoxia include protozooplanktonic (Colpodellida, Picozoa) and parasitic (Peronosporomycetes, Pirsoniales, other) taxa, all of them significantly linked to lower DO and higher temperatures. Similar correlations were found for prokaryotes prevailing in hypoxia: Alphaproteobacteria (Rickettsiales), Gammaproteobacteria (SUP05 and two uncultured lineages), Planctomycetota (Planctomycetales and clade OM190), Verrucomicrobiota (Pedosphaerales), Nitrospinota (Nitrospinales), and one archaean (Nitrosopumilales). Campylobacterota (Sulfurimonadaceae) and Desulfobacterota had a patchier distribution, and only the former correlated significantly with DO. Cyanobacteria (Synechococcales) were widespread and their increased relative abundances during the hypoxic period were significantly linked to higher temperature, but not to lower DO levels.

When combined with previous studies in adjacent LIS waters and other estuaries, our findings add to growing evidence for microbial taxa associated with estuarine hypoxia [21, 57, 78, 79]. These include various prokaryotes (Rickettsiales, SUP05 and OM190 clades, Nitrosopumilales, Nitrospinales, Sulfurimonadaceae, Desulfobacterota) and protistan parasites (Syndiniales, Peronosporomycetes, Pirsoniales, other) that have widespread distributions but appear to be enriched in hypoxic estuaries. While the combined effects of temperature and DO on these taxa are difficult to disentangle, their more frequent or more significant correlations with DO (Fig. 5) and the oxygen-sensitive metabolism of some of them (see next paragraph) suggest a strong linkage between their increased proportions and deoxygenation.

Changes in community structure suggest functional shifts during hypoxia, with potential consequences for nitrogen, sulphur, and carbon biogeochemical cycles. First, the co-occurrence of ammonia-oxidizing archaea (*Nitrosopumilus*) and, in lower proportions, nitrite-oxidizing bacteria (Nitrospinota) exclusively during hypoxia suggests consumption of DO by nitrification, with a likely outpace of ammonia oxidation over nitrite oxidation due to the increased temperatures of late summer [94]. Thus, in addition to heterotrophic oxidation of organic matter, ammonia oxidation is likely a key driver of deoxygenation in the harbour analysed here and in adjacent LIS waters [21]. Second, the detection of sulphide-oxidizing lithoautotrophs (SUP05, Sulfurimonadaceae) and sulphate-reducing organoheterotrophs (Desulfobacterota, possibly associated to particles from resuspended anoxic sediment or forming anoxic microniches [95]) suggests active sulphur cycling during hypoxia. These taxa are among the prevalent sulphur-cyclers in oxygen-deficient eutrophic areas [96] and are linked to sulphide production, although the later was not measured here. Third, the increased prevalence of parasitic taxa during hypoxia, especially Syndiniales, may enhance carbon cycling. Syndiniales parasitize other protists (e.g. Dinophyceae), leading to host lysis and consequent release of organic matter [97]. Enriched pools of organic carbon can then be directly oxidized by heterotrophic prokaryotes or even endocyted by Picozoa [98], another protist group that we found mostly during hypoxia (Fig. S12). Another potential functional shift we did not detect here, but that was apparent in adjacent LIS waters, was a switch from prevalence of large algivorous dinoflagellates in pre-hypoxia to bacterivorous pico- and nanoflagellates in hypoxia [21].

In addition to shifts in taxonomic profiles and potential functions in hypoxia, association networks suggested unique biological interactions during this period (Supplementary Results; Fig. S13, Table S5, Table S6). We detected significant co-occurrences involving at least one partner linked to low DO, including potential host–endosymbiont (Cercozoa–Rickettsiales [99]) and host–parasite (Dinophyceae–Syndiniales [100]) relationships. This suggests that hypoxia generates opportunities for unique connections among taxa favoured under higher temperatures and lower DO levels, specifically symbiotic relationships that may be advantageous to one or both partners.

## Conclusions

Our study demonstrates that summer warming and deoxygenation jointly shape estuarine microbial plankton. We confirm the well-established role of temperature and provide insight into less known DO influences on simultaneously analysed prokaryote and microeukaryote communities. While prokaryote and microconsumer biomass are resilient to deoxygenation, the alpha-diversity and community structure of microbial plankton differ in pre-hypoxic and hypoxic periods, with no effects of other contrasting conditions we analysed (two summers with climate differences; inland-to-open-estuary stations; surface-bottom depths). Our taxonomic profiles in the harbour analysed here and in adjacent, open-estuary waters [21] evidence that hypoxia in the area is driven not only by decomposition of dead phytoplankton blooms, but also by direct ammonia oxidation. Our findings also suggest that, as DO decreases, prokaryotic metabolisms switch from the O_2_-requiring processes that lead to hypoxia (aerobic respiration, nitrification) to anaerobic reductive reactions (e.g. sulphate reduction), while interactions like parasitism and endosymbiosis may be favoured for microeukaryotes. Biological interactions such as viral lysis and grazing have known key roles in structuring microbial communities, with future research needed to quantify changes in biotic relationships under summer warming and deoxygenation. Both warming and deoxygenation act as key regulators of estuarine microbial ecology, food-web dynamics and biogeochemical cycles, thus underscoring their combined importance in forecasting ecosystem responses to ongoing coastal change and under future climate scenarios.

## Supplementary Material

Dharam_et_al_Supplementary_Materials_R1_ycag015

Table_S1_ycag015

Table_S6_ycag015

## Data Availability

The 16S and 18S rRNA gene sequence datasets generated during the current study are available in the NCBI Sequence Read Archive (SRA), accession number PRJNA1329165. The bioinformatic code used to analyse data during this study is available at https://github.com/LuSantof/HempsteadHarbor22-23.
