# Supplementary material for: Summer warming and deoxygenation shape estuarine microbial plankton across domains of life
Source: ISME Commun. 2026 Jan 22;6(1):ycag015. doi: 10.1093/ismeco/ycag015 (PMC12911933; doi:10.1093/ismeco/ycag015)
Supplement: Dharam_et_al_Supplementary_Materials_R1_ycag015 [file dharam_et_al_supplementary_materials_r1_ycag015.pdf]

Summer warming and deoxygenation shape estuarine microbial plankton across domains of life

Jodi Dharam[#], Pukhraj Kaur[#], Amedea Cipriano, Abigail Salgado, Aleena Qureshi, Khabiba Shahid, Carissa Kissoon, Fabian Leija[ʃ], Luciana Santoferrara*

Department of Biology, Hofstra University, Hempstead, NY 11549, USA

[#]Equal contribution

[ʃ]Current affiliation: School of Marine and Atmospheric Sciences, Stony Brook University, Stony Brook, NY 11794, USA

*Corresponding author: Luciana Santoferrara. 114 Hofstra University, Hempstead, NY 11549. Email: luciana.santoferrara@hofstra.edu; ORCID: https://orcid.org/0000-0001-5587-2111

Supplementary Materials

Supplementary Methods 2
Supplementary Results 6
Supplementary Figures 7
Supplementary Tables 20
Supplementary References 26

Supplementary Methods

Study site, sampling and measurement of environmental variables

This study was conducted within Long Island Sound (LIS), a temperate estuary that borders with New York City and its suburbs, where bottom hypoxia occurs yearly due to warmer temperatures, stratification, and oxidation of organic matter fuelled by nutrient enrichment. More specifically, we sampled in Hampstead Harbor, a V-shaped, 5-miles long harbour (Fig. S1). The 14-miles shorelines of the harbour are affected by rainwater runoff from urban and suburban areas, sewage-treatment-plant discharges, and remains of past industries, despite upgrades in wastewater treatment and wetland restoration in the last decades [1]. Bottom-water hypoxia, characterized by dissolved oxygen (DO) < 3.0 mg L^{-1}, and associated negative effects on invertebrates and fish, such as habitat avoidance and mortality are reported in Hempstead Harbor most summers, while anoxic conditions (DO < 1.0 mg L^{-1}) are rare. DO levels between 3.0 and 4.8 mg L^{-1} are considered as suboptimal and are associated to reduced growth and abundance of marine animals in the area [1].

Our study focused on four 2- to 10-m deep stations (St. 6, 1, 3, and 16 from inland to open estuarine waters), approximately equidistant and along the main axis of the harbour (Fig. S1; Table S1). Samplings were conducted from a boat in the summers of 2022 and 2023, about biweekly from June to August (ten dates total), typically between 8 and 10 AM. The most inland station (St. 6) is inaccessible during low tides and was thus sampled less frequently (four dates). Water temperature, salinity, DO, pH, chlorophyll *a* concentration, and turbidity were recorded with a multiparameter reader (Manta+35, Eureka Water Probes, TX, USA in 2022; and EXO2S, YSI, Yellow Springs, OH, USA in 2023). Measurements were done at 0.5 m below the surface (hereafter, surface depth) and at 1-m increments up to 0.5 m above the bottom (hereafter, bottom depth). On each date, quality checks in the first station monitored included a replicated vertical profile of all measured parameters, DO quantifications in bottom samples by the Winkler titration method, and pH testing of surface waters by a wide-range indicator test kit (LaMotte, DE, USA). Chlorophyll *a* records were calibrated against fluorometry quantifications from filtered water samples. Weekly cumulative precipitation prior to each sampling date was estimated with the National Oceanic and Atmospheric Administration's Climate Data Online Tool (https://www.weather.gov/wrh/Climate? wfo=okx).

A total of 68 water samplings were conducted over 10 dates (Table S1). At each station, samples were collected at the surface and bottom depths using a 2.2 L Van Dorn bottle. Whole water was collected into two 120-mL bottles, one empty and one containing sulfuric acid at 0.02 N final concentration (for N quantifications) and a 500-mL amber bottle containing 10 mL of non-acid Lugol's solution at 2% final concentration (for microscopy). Water gently passed through a sieve (250-µm pore size) was collected into duplicate 500-mL amber bottles leaving as little headspace as possible (for flow cytometry and DNA sequencing). All samples were stored in dark, cool conditions until arriving in the laboratory within 1 hour of finalizing the field work. Nitrite, nitrate, ammonia, and total Kjeldahl (ammonia + organic) nitrogen concentrations were determined by colorimetry (EPA protocols 350.1, 351.2 and 353.2; Pace Analytical Services, NY, USA), and these data were also used to calculate total inorganic and organic nitrogen (TIN and TON, respectively).

In the lab, Lugol's samples were stored at 4°C until analysis. The duplicated amber bottles with sieved, non-fixed water were gently homogenized by twenty manual rotations. Aliquots of 14 mL were fixed with P+G fixative (1% paraformaldehyde plus 0.05% glutaraldehyde final concentrations) and stored at -80°C until prokaryote quantification by flow cytometry. The

remaining ~500 mL were filtered through 0.2-μm-pore, 47-mm-diameter polycarbonate membranes (MilliporeSigma, MA, USA). The filtration columns were pre-rinsed at least three times with sample water. Negative filtration controls were generated by passing sterile water through the filtration columns and then a 0.2-μm filter. Filters were placed in 1.5-μL microcentrifuge tubes with 0.5 μL of DNA Buffer (100 mM NaCl, 10 mM Tris-HCl, 25 mM EDTA, and 0.5% SDS, adjusted to final pH 8.0, then sterilized with a 0.2-μm filter) and stored at -20°C until DNA extraction.

Abundance and biomass of microbial plankton

The abundance of prokaryotes was estimated by flow cytometry within 2-3 months of sample collection. The P+G samples were thawed in the dark at 37°C for about 5 min and homogenized by gentle rotation. Immediately after, samples were diluted 1/10 in MilliQ water and stained with SYBR Green I (Life Technologies, CA, USA) at a 10x final concentration in the dark for 10 min [2]. Yellow-green, 1-μm FluoSphere beads (Life Technologies, CA, USA) were added as a reference for florescence and size. Cell counts were done with a FACSVerse flow cytometer and FACSuite software (Becton, Dickinson and Company, NJ, USA). Events were discriminated according to their green fluorescence (FICT) and side scatter (SSC) properties. Cell detections were converted into abundances (cells mL^{-1}), using flow rates previously estimated by high-precision weighs of MilliQ water samples before and after passage through the instrument for known periods of time [3]. To estimate prokaryote biomass (μg C L^{-1}), a conversion factor of 20 fg C $cell^{-1}$ [4] was applied. While this conversion factor is limited in that it does not consider cell size or taxonomic variations in C content, it yields a linear relationship to abundance and the obtained biomass values are thus used here for statistical analyses analogous to the ones described below for microconsumers; we do avoid comparisons of prokaryote and microconsumer biomass values given that they were obtained with substantially different methods, each of them presenting their own limitations [5].

Within microeukaryotes, we estimated the abundance of consumer organisms (referred as to microconsumers from now on) by inverted microscopy [6]. We focused on primarily consumers (i.e. those that cannot grow without consuming other organisms: protozooplankton, other zooplankton and non-constitutive mixoplankton = NCM) in the ~20-200 μm size fraction. This included aloricate and loricate ciliates, dinoflagellates (only species known to be heterotrophs and NCMs; [7, 8]), and micrometazoans (copepod nauplii and rotifers). Flagellates <20 μm were not resolved by our method. Aloricate ciliates in the genus *Mesodinium* (mostly *M. rubrum* and *M. major*) were not included in the microconsumer category, given that these NCMs are strongly photosynthetic and considered analogous to constitutive mixoplankton [7]. The 500-mL Lugol's samples were settled down in graduated cylinders for 24 h, then concentrated down to 50 mL by aspiration of the supernatant [9]. The concentrate was homogenized by 20 rotations, aliquots of 5 mL were settled down, and at least 200 (median = 623) specimens per sample were counted in a DMi8 inverted microscope (Leica, IL, USA) at 200X total magnification. Specimens were classified based on shape and size, and random individuals were measured for biovolume estimates using the LAS X software (Leica, IL, USA). To estimate biomass, biovolume-to-C conversion factors were applied for protists [10-12], and length-based regressions were used for micrometazoans [13, 14]. The abundance of primarily photosynthetic organisms (phytoplankton plus constitutive mixoplankton) was not determined, and instead we used chlorophyll *a* concentration (described above) as a rough indicator of their biomass.

Community DNA extraction, library construction and sequencing
DNA was extracted with the Fecal/Soil Microbiome kit D6012 (Zymo Research, CA, USA). Negative extraction controls (lysis buffer) were generated to check for contamination during laboratory procedures. DNA samples were quantified with the Qubit DNA broad range assay (Life Technologies, CA, USA).

The extracted DNA was used to separately amplify the V4 regions of the 16S and 18S rRNA genes (referred as to 16S or 18S from now on), using the 515F - 806R primers [15, 16] for prokaryotes and the TAReuk454FWD1 - TAReukREV3 primers [17] for microeukaryotes, respectively. The 16S primers were customized for Illumina dual indexing of amplicons in a single PCR step [18]. The 18S primers included an overhang, and a second PCR was needed for dual indexing [19]. Reactions included 10 ng of DNA and the following reagents (Promega GoTaq® PCR Core Systems, WI, USA): 5.0 µl 5X buffer, 2.5 µl $MgCl_2$, 0.7 µl dNTPs (10 mM), 0.25 µl Taq Polymerase, and 1 µl of each primer (10 µM). For 16S, PCR conditions were: 95°C for 3.5 min, 30 cycles of 95°C for 30 s, 50°C for 30 and 72°C for 90 s, and final extension at 72°C for 10 minutes. For 18S, conditions for the first PCR were: 95°C for 2 min, 10 cycles of 95°C for 10 s, 53°C for 30 s and 72°C for 30 s, followed by 15 cycles of 95°C for 10 s, 48°C for 30 s and 72°C for 30 s, and final extension at 72°C for 2 min; and for the second PCR: 95°C for 3.5 min, 8 cycles of 95°C for 30 s, 50°C for 30 s and 72°C for 90 s, and final extension at 72°C for 10 min.

Triplicate PCR products per sample were pooled before quantification and visualization with the QIAxcel DNA Fast Analysis (Qiagen, Hilden, Germany). Pools were normalized based on expected amplicon length and DNA concentration, then combined with the QIAgility liquid handling robot, and cleaned with the Gene Read Size Selection kit (Qiagen, Hilden, Germany). Paired-end sequencing was done with the MiSeq instrument and the 2x250 base pair kit v. 2 (Illumina Inc., CA, USA) at the MARS (Microbial Analysis, Resources and Services) facility, University of Connecticut. The negative filtering and extraction controls were amplified with both primer sets and sequenced along with the samples. These negative controls yielded a negligible number of reads and sequence compositions significantly different from the samples. The ZymoBIOMICS Microbial Community DNA Standard D6305 / D6306 (Zymo Research, CA, USA) was amplified with the 16S primer set, sequenced, and checked for the expected even distribution of amplicon relative abundances. A total of 11.5 million 16S and 18S reads were generated (N = 66 and 68 samples, respectively, as two samples failed for 16S) and the raw datasets are available in NCBI's Sequence Read Archive (accession number PRJNA1329165).

Processing of raw DNA sequences
DNA sequences were demultiplexed and quality-filtered with BaseSpace (Illumina Inc., San Diego, CA). Additional sequence processing was done in QIIME 2 [20], including primer trimming with the cutadapt plugin [21] and denoising, dereplication, and chimera removal with DADA2 [22]. Taxonomic classification of amplicon sequence variants (ASVs) used a Naive Bayes classifier [23] trained on V4-trimmed versions of SILVA v. 138 for 16S [24] and PR2 v. 5.0.0 for 18S [25, 26]. ASVs annotated as chloroplast, mitochondrion, or non-target sequences were eliminated from both datasets. Microeukaryote taxa were annotated into trophic groups (phytoplankton = exclusively photo-trophic protists; protozooplankton = exclusively phago-trophic protists; constitutive and non-constitutive mixoplankton = photo-phago-trophic protists that produce chloroplasts or acquire photosynthetic capacity from their prey, respectively;

parasitic protists; and micrometazoans) based on trophic strategy information available in metaPR2 [27].

Data analysis

Analyses conducted during this study are detailed in the main text of this publication. Additional analyses included an alternative alpha-diversity metrics, Faith's phylogenetic diversity index [28], which considers variant richness and their phylogenetic relatedness (the sum of the lengths of variant branches on a tree built with MAFFT-aligned variants and FastTree under default parameters as implemented in QIIME2; [20]). We also analysed association networks among bacteria, archaea and microeukaryotes, which were built with SpiecEasi [29] and visualized with iGraph [30] as implemented in the NetCoMi R package v.1.2 [31]. The non-subsampled 16S and 18S datasets were agglomerated into orders and samples were grouped into pre-hypoxic and hypoxic periods. The 100 most prevalent orders per dataset and period were selected. SpiecEasi, a compositionally-aware conditional independence method that handles zeros via model-based approaches and applies a centred log-ratio transformation, was used with neighbourhood selection, nlambda = 20, sparsification threshold = 0.4, and the stability approach to regularization selection [29]. For visualization, only taxa present in each period were retained in the networks, and unconnected nodes were removed. Statistical differences between the pre-hypoxia and hypoxia networks were assessed with non-parametric permutation tests using 1000 iterations [31].

Supplementary Results

Association networks

Association networks among prokaryote and microeukaryote taxa presented general similarities during pre-hypoxia and hypoxia (Fig. S12, Table S5). In both periods, there were more and stronger positive connections (co-occurrences) than negative ones, and both types of connections were more frequent within than between domains. Bacteria and microeukaryotes had a similar number of nodes in both networks, while the presence of only one Archaea order in each network relates to the lower relative abundances of this domain in our data.

Still, the pre-hypoxia and hypoxia networks differed significantly in metrics related to the number of connections between orders and how orders group together in the networks (degree and betweenness centrality and differences in clustering; see Table S5 for interpretations). Among hubs (nodes potentially more influential within the network structure; [31]), only Flavobacteriales matched between pre-hypoxia and hypoxia, suggesting that this order is key in the network structure over the summer and independently of temperature or DO changes. Flavobacteriales, and other associated nodes (e.g., Cellvibrionales and Oceanospirillales) are important degraders of organic matter produced by phytoplankton like diatoms and dinoflagellates [32, 33], although such cross-domain relationships were not detected in the networks. Likewise, other expected relationships remained undetected, for example between *Nitrosopumilus* and Nitrospinota during hypoxia (due to the low proportion and thus exclusion of the later order from the network). Some observed associations that may indicate biological interactions include potential predator–prey (e.g., Colpodellida – Cryptomonadales [34]), host–endosymbiont (e.g., Rickettsiales – Cercozoa [35]) and host–parasite (Syndiniales – Dinophyceae [36]) partners, and such connections were detected exclusively during hypoxia (Fig. S12). This suggests that the hypoxic period generate opportunities for unique connections among taxa favored under higher temperatures and lower DO levels, specifically symbiotic relationships that may be advantageous to one or both partners.

Supplementary Figures

Figure S1. Long Island Sound. The study area is enlarged and show station location (blue circles). Maps built with Ocean Data View v5.7.2 (https://odv.awi.de).

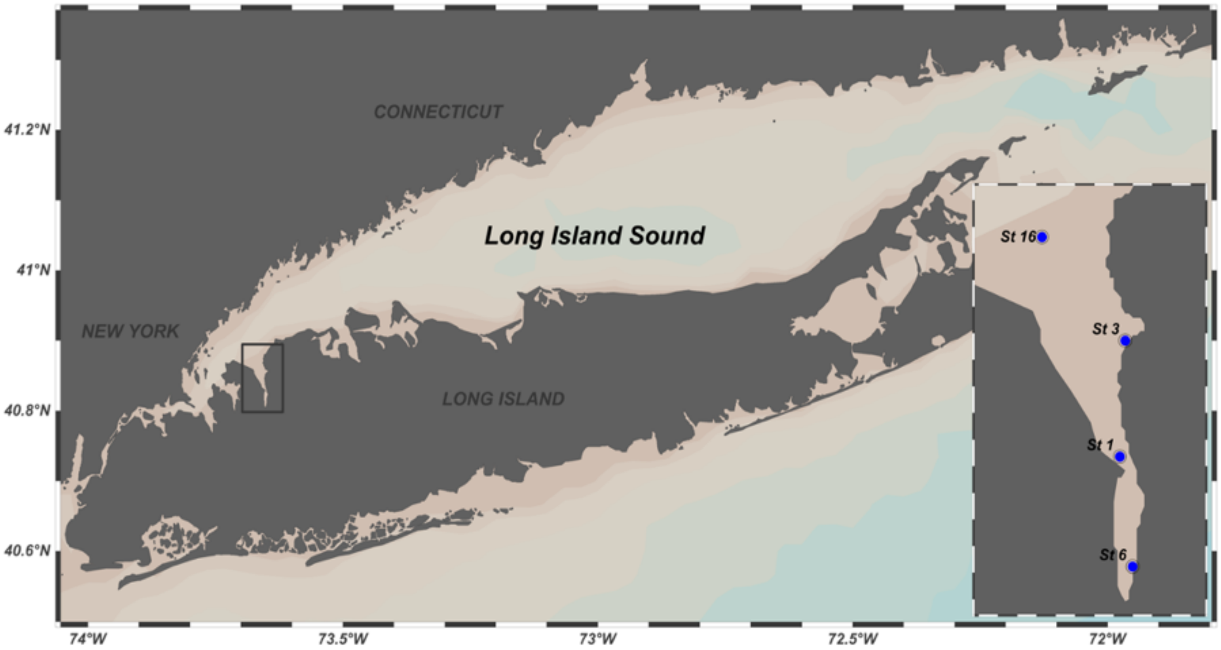

Figure S2. Environmental variables (additional to those shown in Fig. 1). **A** Salinity, **B** surface-water dissolved oxygen (DO), **C** pH and **D** Turbidity per date during pre-hypoxia (PreH) and hypoxia (H) 2022 and 2023 (N = 68 in A, C, and D including both surface and bottom waters; N = 34 in B). Boxplots show medians, interquartile ranges (IQR, boxes), ranges within 1.5 times the IQR (whiskers), and outliers. In B, dotted lines indicate hypoxic and suboptimal DO thresholds. Kruskal-Wallis tests among periods were significant (p-value < 0.01) for all four variables; Dunn's comparisons were significant (p-value < 0.01) for all four variables between pre-hypoxic and hypoxic periods within each year. Nitrate, nitrite, ammonia, TIN, and TON were excluded given that most values were below the detection limit (Table S1).

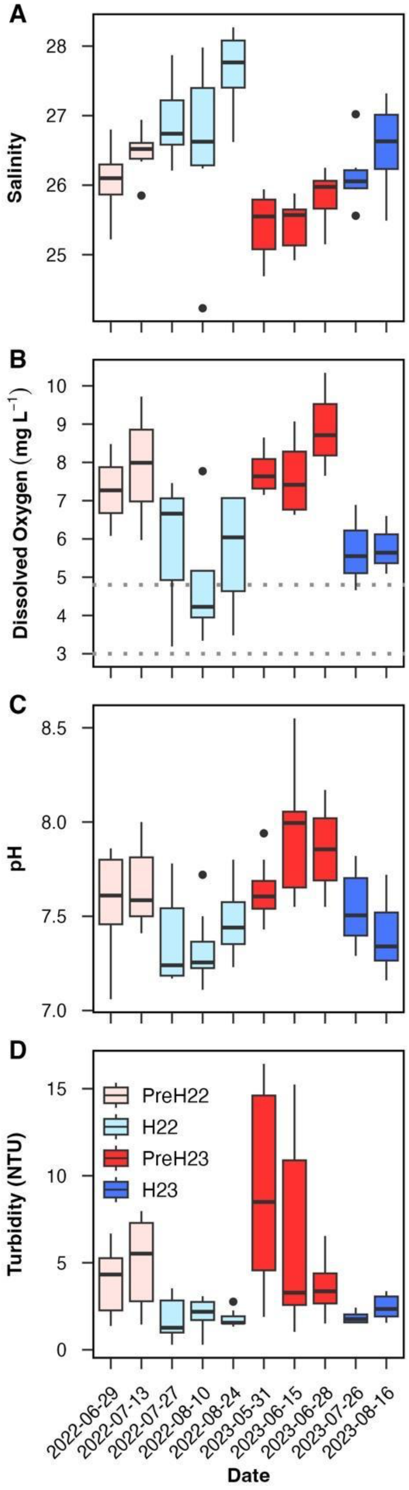

Figure S3. Abundance. **A** Prokaryote and **B** microconsumer abundance values per date during pre-hypoxia (PreH) and hypoxia (H) 2022 and 2023 (including both surface and bottom waters; N = 34 in A as not data is available for 2022; N = 64 in all other graphs). **C-G** Abundance of each microconsumer group (including aloricate and loricate ciliates, microconsumer dinoflagellates and micrometazoans). Aloricate ciliates exclude those in the genus *Mesodinium* (see Supplementary Methods). Boxplots show medians, interquartile ranges (IQR, boxes), ranges within 1.5 times the IQR (whiskers), and outliers.

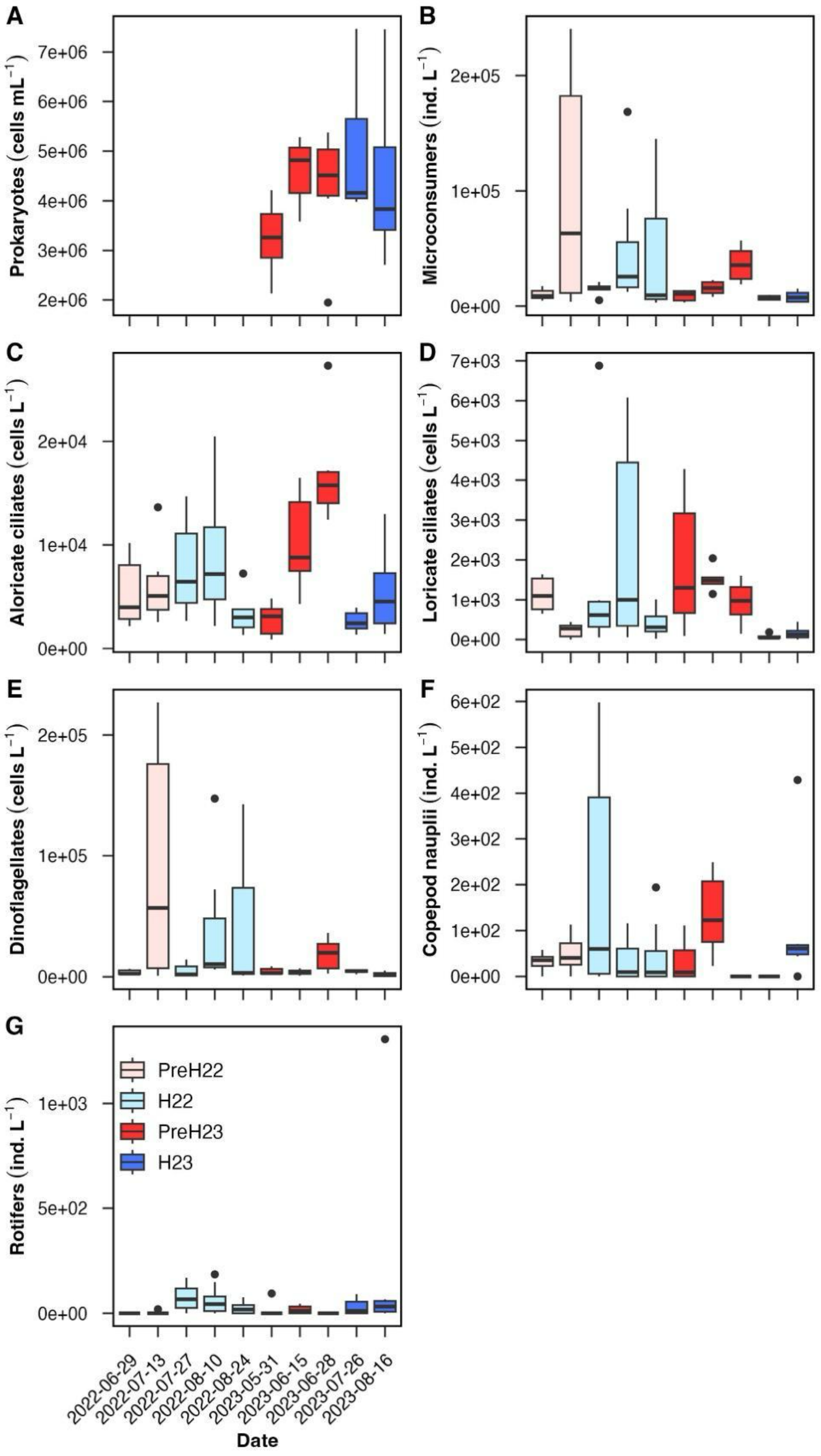

Figure S4. Biomass. **A** Prokaryote and **B** microconsumer biomass values per date during pre-hypoxia (PreH) and hypoxia (H) 2022 and 2023 (including both surface and bottom waters; N = 34 in A as not data is available for 2022; N = 64 in all other graphs). **C-G** Biomass of each microconsumer group (including aloricate and loricate ciliates, microconsumer dinoflagellates and micrometazoans). Aloricate ciliates exclude those in the genus *Mesodinium* (see Supplementary Methods). Boxplots show medians, interquartile ranges (IQR, boxes), ranges within 1.5 times the IQR (whiskers), and outliers.

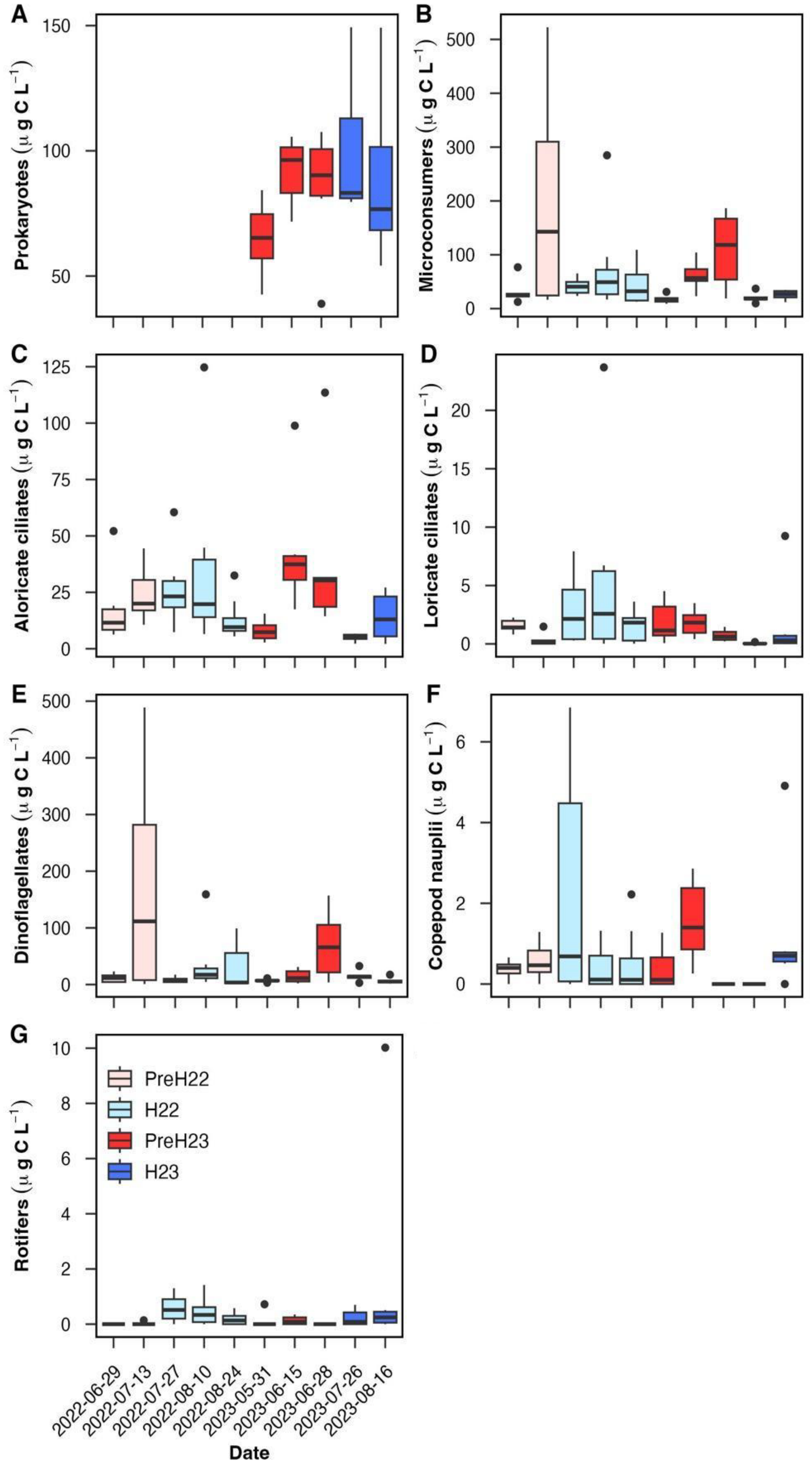

Figure S5. A Relationship between the biomass of microconsumer dinoflagellates (only taxa known as protozooplankton and non-constitutive mixoplankton) and chlorophyll *a* concentration. The maximum value for both variables was recorded in Station 1, surface, on 7/13/2022. **B** In this sample, microscope observations indicated prevalence of dinoflagellates belonging to protozooplankton (*Polykrikos*, *Gyrodinium*) and chlorophyll-containing (*Prorocentrum)* genera. Microscope observations were confirmed by DNA metabarcoding results for this sample, including prevalence of *P. kofoidii, G. dominans, G. dorsalisulcum*, and *G. spirale* as protozooplankton representatives. In terms of chlorophyll-containing species, metabarcoding results showed prevalence of unresolved *Prorocentrum spp*. (with ASVs presenting 100% matches to the constitutive mixoplankton (CM) *P. redfieldii* and phytoplanktonic *P. triestinum* and *P. gracile*; of them, *P. triestinum* is usually reported in Long Island Sound based on microscopy, although differentiation from *P. redfieldii* is challenging; [37]), as well as three nanoplanktonic species: the phytoplanktonic dinoflagellate *Biecheleria brevisulcata*, the CM dinoflagellate *Heterocapsa rotundata,* and the CM, harmful-algal-bloom-forming Raphidophyceae *Heterosigma akashiwo*.

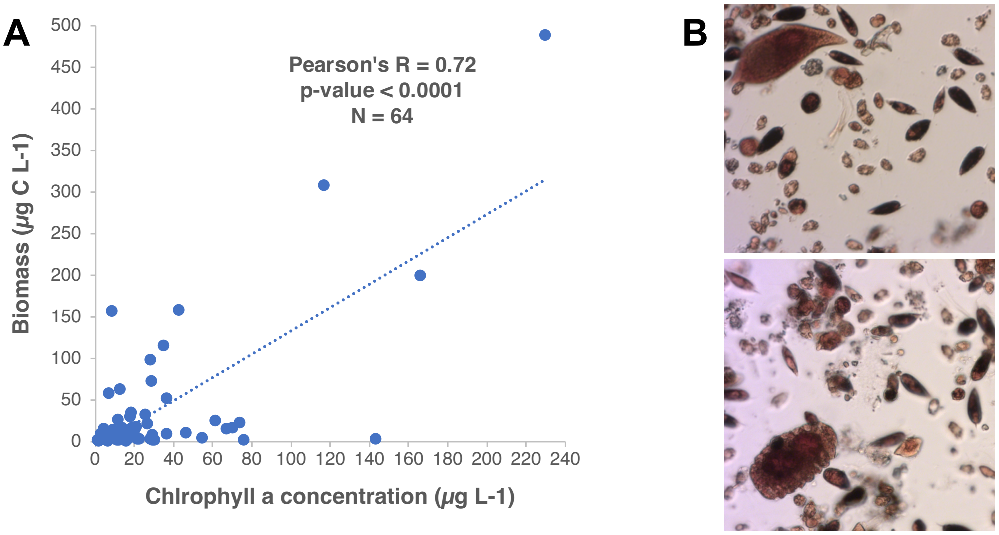

Figure S6. Our study captured most alpha-diversity and estimates were not affected by sub-sampling. **A, B** Prokaryotes (N = 66) and **C, D** microeukaryotes (N = 68). Shannon index values increased at sampling depths up to 2,000-3,000 reads and remained almost invariable beyond the selected subsampling levels (dashed lines) both per date (**A, C**) and during pre-hypoxia (PreH) and hypoxia (H) 2022 and 2023 (**B, D**). Marginal random effects were observed for iterations close to the subsampling depths.

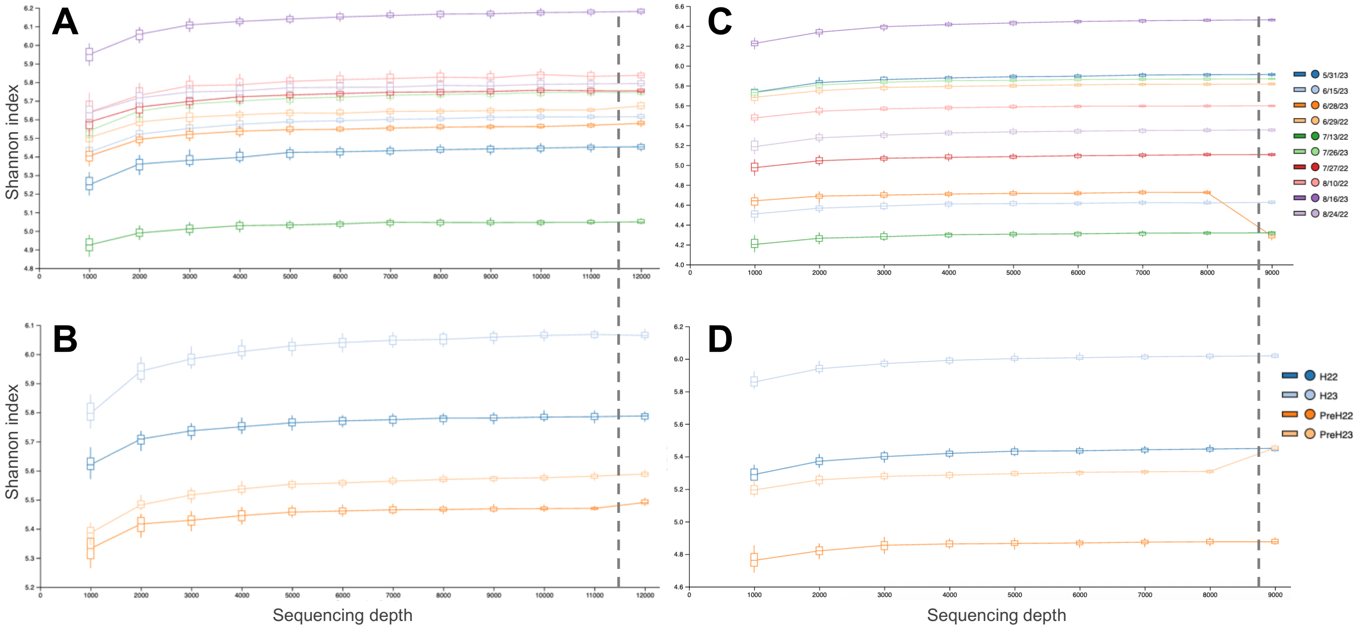

Fig. S7. Generalized additive model (GAM) results for prokaryote Shannon index additional to those shown in Fig. 3. GAMs as detailed in Fig. 3. Additional GAM diagnostics are provided in Table 2.

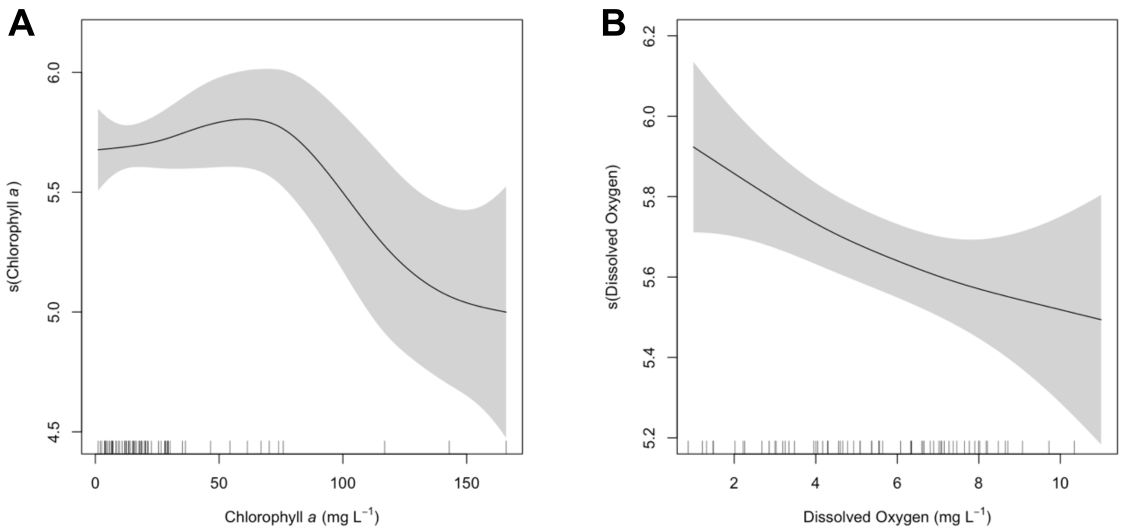

Fig. S8. Alpha-diversity based on Faith's phylogenetic diversity index. A Prokaryote and C microeukaryote values during pre-hypoxia (PreH) and hypoxia (H) in 2022 and 2023 (including both surface and bottom waters; N = 66 in A; N = 68 in C). Boxplots as detailed in Fig. 1. Kruskal-Wallis tests among periods were significant (p-value < 0.01); asterisks indicate Dunn's test significance (p-value < 0.05* or < 0.01**) for comparisons within each year. B and D Generalized additive model (GAM) results for prokaryote (B) and microeukaryote (D) Shannon index. GAMs as detailed in Fig. 2; only the most significant smooth functions are included (p < 0.05).

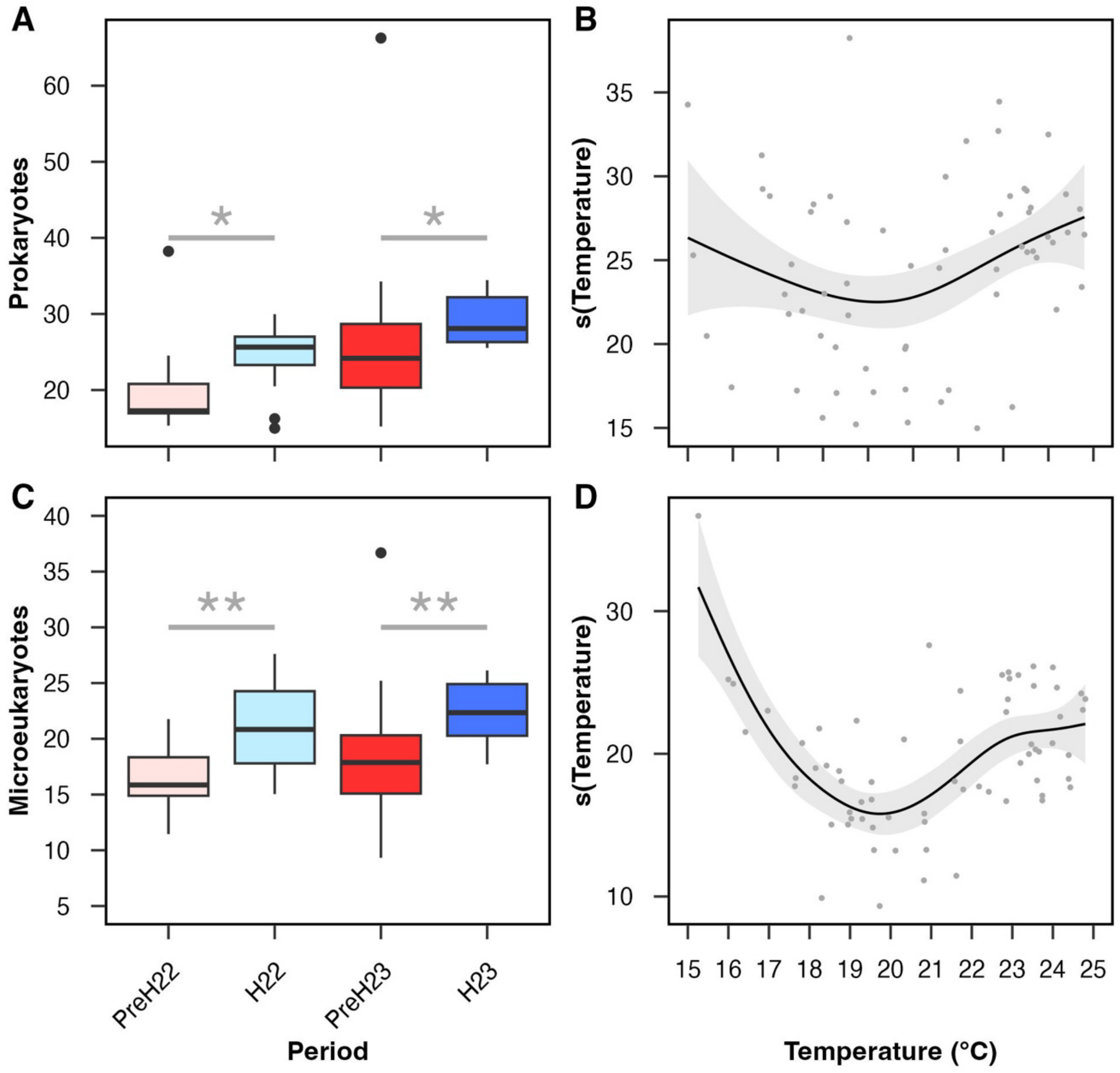

Fig. S9. **A** Prokaryote and **B** microeukaryote principal coordinates analyses (PCoA) identical to those displayed in Fig. 4 but showing three axes (instead of two) and with samples coloured by sampling date (instead of period). Adding a third axis in the PCoA plots did not change the ordination of samples, but incorporated explained variance to the analyses, which totals 56% and 37% for prokaryotes and microeukaryotes, respectively. Colour gradients in sample makers and arrows indicate progression from the first (clearer) to the last (darker) sampling date in 2022 (purple palette) and 2023 (green palette).

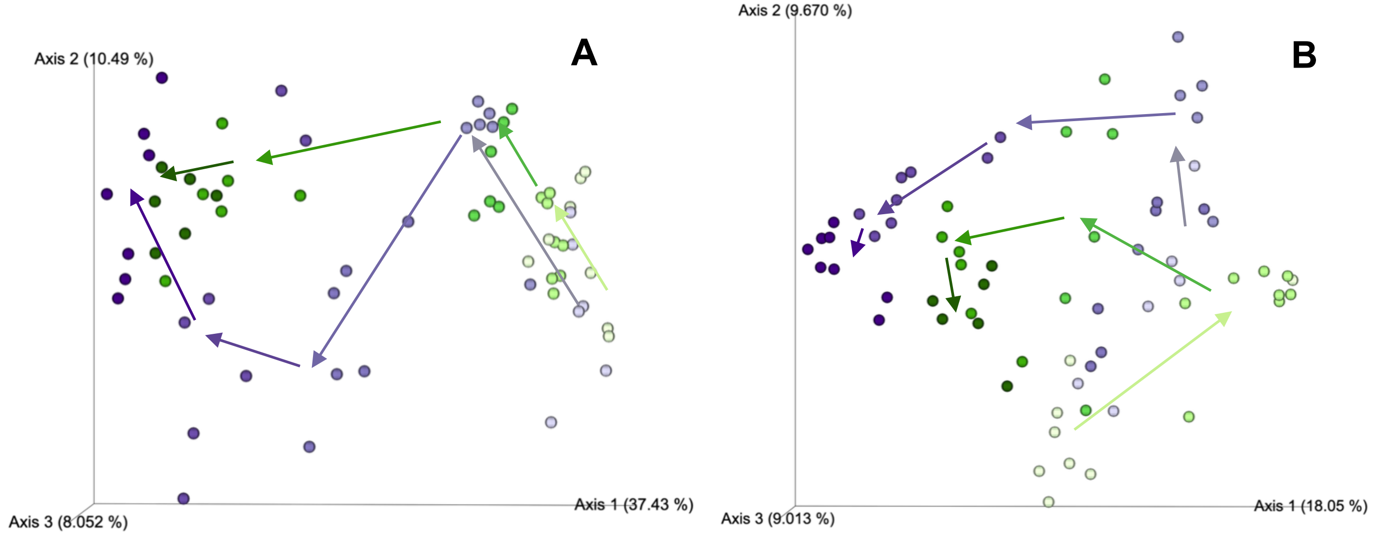

Figure S10. **A** Prokaryote and **B** microeukaryote principal coordinates analyses (PCoA) based on Aitchison distance matrices (Euclidean distances based on centered log-ratio transformed total reads and added pseudo-count of 1 for zeros). Note that in these circumstances, a PCoA is equivalent to a principal component analysis (PCA). Results were similar to those shown in Fig. 4, but here at least the 16S results are possibly affected by the unequal number of total reads obtained in 2022 and 2023 (double in 2023 for 16S where we see the bias, while almost equal in both years for 18S, where no bias is apparent). Pink = pre-hypoxia 2022, light blue = hypoxia 2022, red = pre-hypoxia 2023, blue = hypoxia 2023.

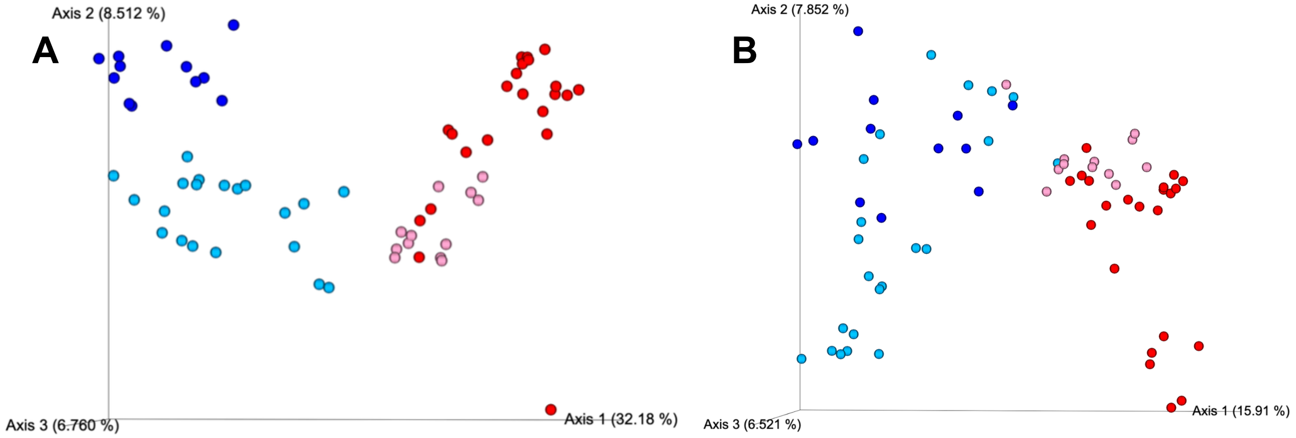

Figure S11. Heatmap based on 16S read proportions during pre-hypoxia (PreH) and hypoxia (H) 2022 and 2023 (N = 66). The heatmap was built with the qiime2R package [38] based on subsampled reads then converted to relative contributions. The four horizontal boxes group taxa based on their distribution and prevalence: widespread and prevalent (present in all or most samples and contributing >2% of the total reads); widespread but not prevalent (same but contributing 2% or less to the total reads); prevailing during pre-hypoxia; and prevailing during the hypoxic period (SUP05, Desulfobacterota, Cyanobacteria and OM190 were widespread but more prevalent in hypoxia). Taxonomic names match the used reference database (SILVA v.138; [24]) for reproducibility. All taxa are Bacteria, except one Archaea (A_). ASVs were manually grouped into taxonomic ranks that retain the most informative categories (in most cases, phylum_order or class_order; two families and one genus were also retained; well-known order- or family-level uncultured clades were retained; two uncultured lineages include their highest Blast match in the names).

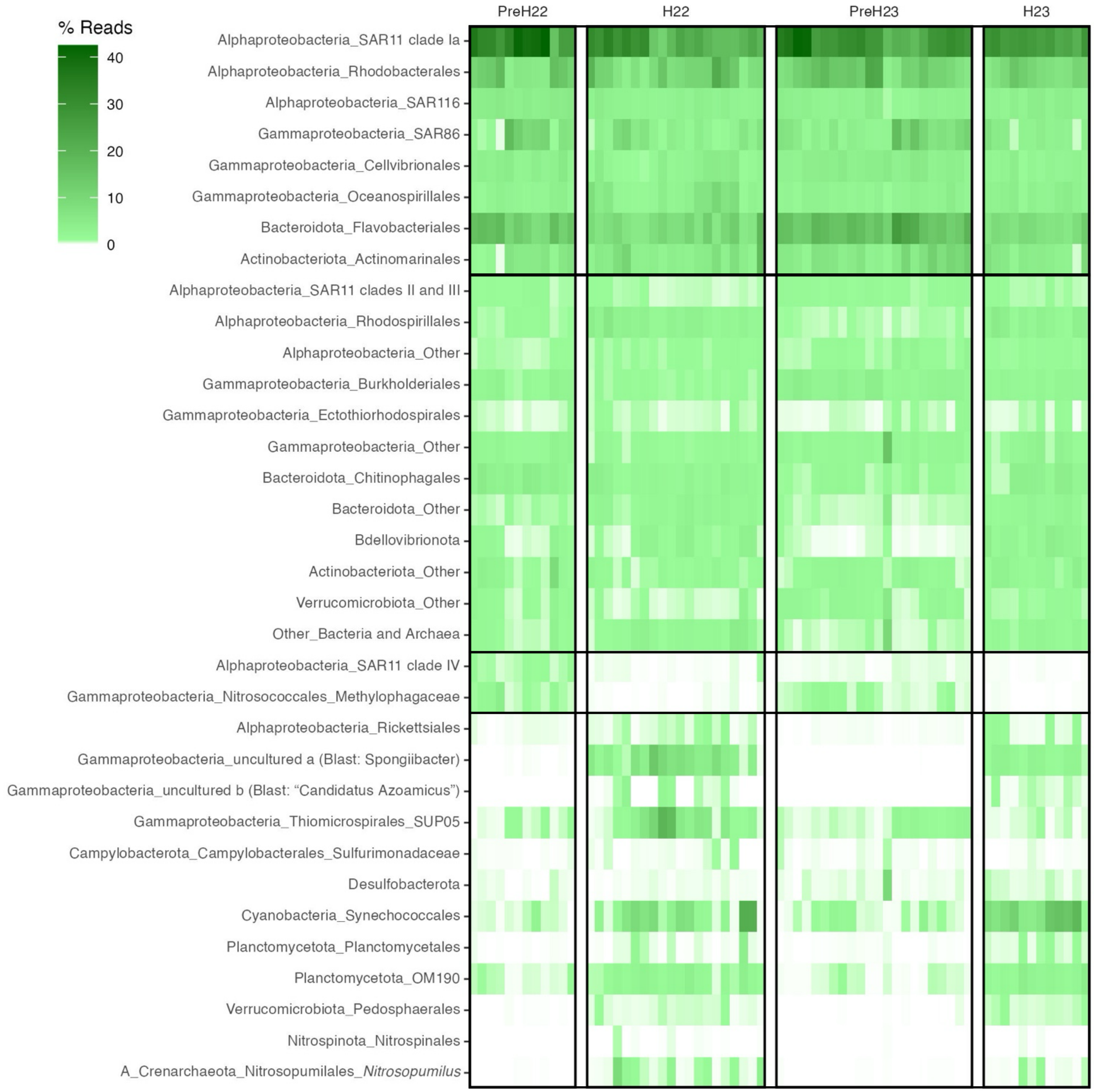

Figure S12. Heatmap based on 18S read proportions (% reads) during pre-hypoxia (preH) and hypoxia (H) in 2022 and 2023 (N = 68). The heatmap was built with the qiime2R package [38] based on subsampled reads then converted to relative contributions. The four horizontal boxes group taxa based on their distribution and prevalence: widespread and prevalent (present in all or most samples and contributing >2% of the total reads); widespread but not prevalent (same but contributing 2% or less to the total reads); prevailing during pre-hypoxia; and prevailing during the hypoxic period (the latter two categories include phytoplanktonic and parasitic Dinoflagellata, which were widespread but more prevalent in pre-hypoxia or hypoxia, respectively).

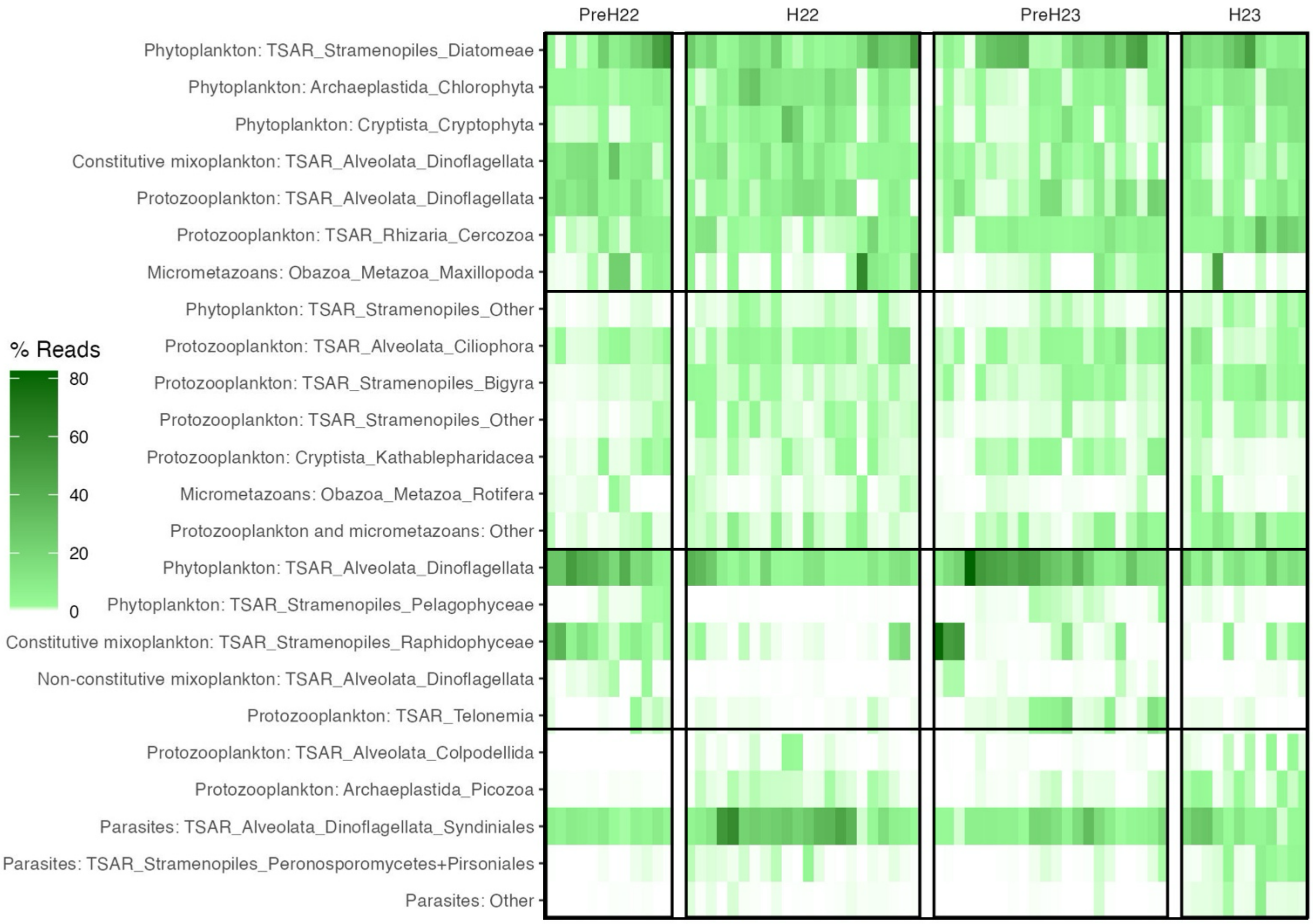

Figure S13. Association networks combining 16S and 18S data collected in 2022 and 2023. **A** Pre-hypoxic (N = 34) and **B** hypoxic (N = 32) periods. Each node represents an order; node size is proportional to the number of reads after a centred log-ratio transformation; numbers on each node indicate the order name (as detailed in Supplementary Table S6); node colours indicate domain affiliation (Bacteria = yellow, Archaea = blue, Eukarya = pink). Nodes with a thicker green border are “hubs” (based on eigenvector centrality, which identifies key nodes based on number of connections and how well-connected the neighbour nodes are). Nodes with no connections were not plotted. Each edge (connection) between nodes represents a positive (red) or negative (blue) association; edge thickness indicates edge weight. Only the 100 most prevalent orders per dataset (16S or 18S) and period (pre-hypoxia or hypoxia) were included. Some nodes of interest include 3 (Nitrosopumilales), 7 (Woesearchaeales), 82 (Flavobacteriales), 164 (Cellvibrionales), 165 (Oceanospirillales), 218 (SAR116), 24 (Rickettsiales), 377 (Cercozoa: Ventricleftida), 532 (Colpodellida), 449 (Cryptomonadales), 529 (Syndiniales: Dino-Group-II), 540 (Dinophyceae: Gymnodiniales).

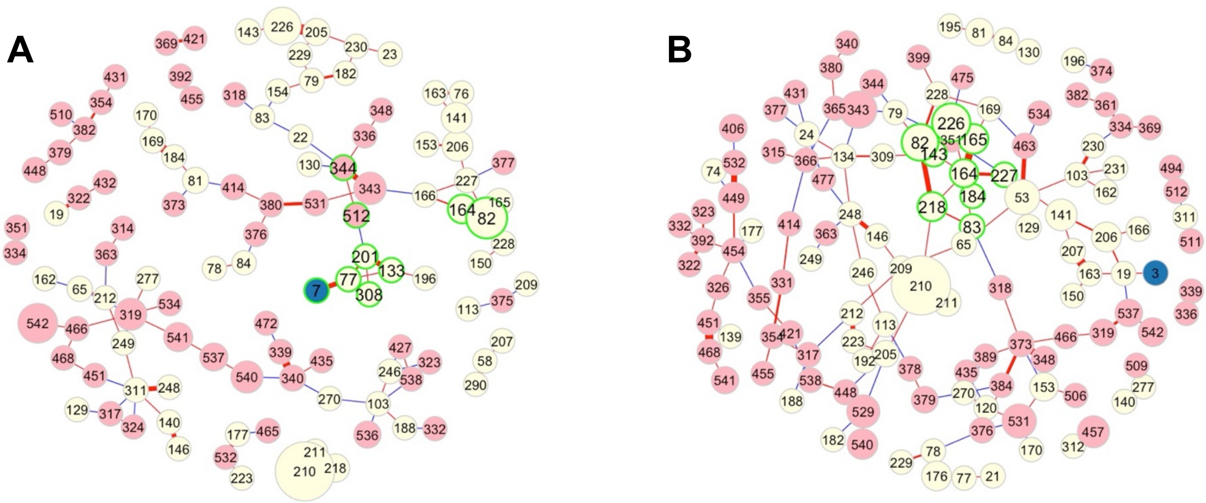

Supplementary Tables

Table S1. Sampling metadata. Dates were classified into pre-hypoxic (PreH) and hypoxic (H) periods based on general conditions in the area [1]. Table provided as a separate .xlsx file.

Table S2. Correlations among environmental variables. Person's R coefficients are shown (N = 68). Values in bold are significant after applying the Benjamini-Hochberg adjustment for multiple testing (*p-value <0.05; **p-value <0.01). Nitrate, nitrite, ammonia, total inorganic nitrogen and total organic nitrogen were excluded given that most values were below the detection limit (Table S1).

	Sample Depth (m)	Temperature (°C)	Dissolved Oxygen (mg L^{-1})	Salinity	pH	Chlorophyll *a* (µg L^{-1})
Temperature (°C)	**-0.31***					
Dissolved Oxygen (mg L^{-1})	**-0.53****	**-0.30***				
Salinity	**0.45****	**0.41****	**-0.62****			
pH	**-0.33***	-0.27	**0.82****	**-0.47****		
Chlorophyll *a* (µg L^{-1})	**-0.30***	0.08	0.13	-0.15	-0.08	
Turbidity (NTU)	0.18	**-0.58***	0.14	**-0.39****	0.04	0.15

Table S3. Median (standard deviation) values per period for variables shown in Fig. 3A, C and 4A, C. N = total number of samples. N.A. = not available.

Period	N	Prokaryote biomass (μg C L^{-1})	Microconsumer biomass (μg C L^{-1})	Prokaryote Shannon index	Microeukaryote Shannon index
Pre-hypoxia 2022	12	N.A.	26.86 (164.84)	5.47 (0.49)	4.86 (0.91)
Hypoxia 2022	22	N.A.	35.82 (57.79)	5.81 (0.36)	5.43 (0.64)
Pre-hypoxia 2023	22	83.14 (20.26)	46.62 (56.44)	5.60 (0.57)	5.32 (1.10)
Hypoxia 2023	12	83.01 (31.16)	21.04 (9.09)	6.08 (0.26)	6.04 (0.57)

Table S4. Mantel correlation coefficients (R) and BIOENV ranks. Out of all possible combinations of environmental factors, the highest correlation with the Bray-Curtis dissimilarity matrix was given by only one variable (temperature) for both prokaryotes and microeukaryotes.

Variables	Rank	R (prokaryotes, N = 66)	R (microeukaryotes, N = 68)
Temperature (Temp)	1	0.653	0.479
Temp + Salinity (Sal)	2	0.651	0.470
Temp + Sal + Dissolved oxygen (DO)	3	0.603	0.449
Temp + Sal + DO + pH	4	0.534	0.444[a]
Temp + Sal + DO + pH + Turbidity (Tur)	5	0.500	0.438[b]
Temp + Sal + DO + pH + Tur + Sample Depth (Depth)	6	0.442	0.408
Temp + Sal + DO + pH + Tur + Depth + Chlorophyll *a*	7	0.379	0.339

[a]Includes turbidity instead of pH; [b]includes sample depth instead of pH.

Table S5. Differences between the pre-hypoxia and hypoxia association networks illustrated in Fig. S13. Significant values (p-value < 0.05) and matching hubs in pre-hypoxia and hypoxia are shown in bold. A = Archaea; B = Bacteria; E = Eukarya. N.A. = not applicable.

**Metric**	**Interpretation**	**Statistic**	**p-value**
**1. Differences between most central nodes**			
Degree centrality	Number of connections (edges) between nodes	0.140	**0.002**
Betweenness centrality	Number of connections between groups of nodes (clusters)	0.177	**0.005**
Closeness centrality	Top nodes based on shortest paths to all others	0.235	0.053
Eigenvector centrality	Top nodes based on number of connections and how well-connected the neighbor nodes are	0.254	0.103
**2. Differences in clustering**			
Whole network	How nodes group together within the full network	0.050	**0.009**
Largest Connected Component	How nodes group together within the largest connected subnetwork	0.051	**0.000**
**3. Differences in global network properties**			
Clustering coefficient	Tendency of nodes to form clusters	0.121	**0.042**
Modularity	Strength of clustering	0.065	0.374
Positive edges	Proportion of positive associations among taxa	1.254	0.891
Edge density	Proportion of actual connections out of all possible connections	0.002	0.308
Average path length	Average number of steps between nodes	0.907	0.473
**4. Hubs**			
Hubs in Pre-hypoxia:	Hubs in Hypoxia:	N.A.	N.A.
7_A_Nanoarchaeota_Woesearchaeales	**82_B_Bacteroidota_Flavobacteriales**	N.A.	N.A.
77_B_Bacteroidota_Bacteroidales	83_B_Bacteroidota_Balneolales	N.A.	N.A.
**82_B_Bacteroidota_Flavobacteriales**	143_B_Actinobacteriota_Micrococcales	N.A.	N.A.
133_B_Desulfobacterota_Desulfobulbales	164_B_Proteobacteria_Gamma_Cellvibrionales	N.A.	N.A.
164_B_Proteobacteria_Gamma_Cellvibrionales	165_B_Proteobacteria_Gamma_Oceanospirillales	N.A.	N.A.
201_B_Desulfobacterota_Desulfobacterales	184_B_Proteobacteria_Gamma_Tenderiales	N.A.	N.A.
308_B_Verrucomicrobiota_Omnitrophales	218_B_Proteobacteria_Alpha_Puniceispirillales	N.A.	N.A.
344_E_Gyrista_Chaetocerotales	226_B_Proteobacteria_Alpha_Rhodobacterales	N.A.	N.A.
512_E_Ciliophora_Oligotrichida	227_B_Proteobacteria_Alpha_Parvibaculales	N.A.	N.A.

Table S6. Numerical reference for orders used in association networks. Note that Fig. S13 only includes the 100 most prevalent orders per dataset (16S and 18S) and period (pre-hypoxia and hypoxia). Taxonomic ranks used for prokaryotes (phylum, class, order) do not always apply to eukaryotes; thus, the most approximate ranks available in PR2 (ranks 4, 5 and 6) are shown for eukaryotes. Ranks with no assignment were propagated from the adjacent taxon. Table provided as a separate .xlsx file.

Supplementary References

1. Coalition to Save Hempstead Harbor, *2023 Water-Quality Report, Hempstead Harbor*. 2024. p. 268 pp.
2. Marie, D., et al., *Enumeration and cell cycle analysis of natural populations of marine picoplankton by flow cytometry using the nucleic acid stain SYBR Green I.* Appl. Environ. Microbiol., 1997. **63**: p. 186–193.
3. Gasol, J.M. and X.A.G. Morán, *Flow Cytometric Determination of Microbial Abundances and Its Use to Obtain Indices of Community Structure and Relative Activity*, in *Hydrocarbon and Lipid Microbiology Protocols*. 2015. p. 159-187.
4. Lee, S. and J.A. Fuhrman, *Relationships between Biovolume and Biomass of Naturally Derived Marine Bacterioplankton.* Appl Environ Microbiol, 1987. **53**: p. 1298-1303.
5. Alder, V. and C. Morales, *Manual de métodos para el estudio de sistemas planctónicos marinos*. 2009, Buenos Aires: EUDEBA. 272.
6. Uthermöhl, H., *Zur Vervollkommung der quantitativen Phytoplankton-Metodik.* Mitt. Int. Ver. Theor. Angew. Limnol., 1958. **9**: p. 1-38.
7. Mitra, A., et al., *The Mixoplankton Database (MDB): Diversity of photo-phago-trophic plankton in form, function, and distribution across the global ocean.* J Eukaryot Microbiol, 2023. **70**(4): p. e12972.
8. Kraberg, A., M. Baumann, and C. Dürselen, *Coastal Phytoplankton. Photo Guide for Northern European Seas.* 2010, Munchen: Verlag Freidrich Pfeil. 204.
9. Claessens, M. and M. Prast, *Concentration of fixed plankton samples via settling: how long is long enough?* J. Plankton Res., 2008. **30**(1): p. 57-64.
10. Putt, M. and D. Stoecker, *An experimentally determined carbon: volume ratio for marine "oligotrichous" ciliates from estuarine and coastal waters.* Limnol. Oceanogr., 1989. **34**(6): p. 1097-1103.
11. Verity, P. and C. Langdon, *Relationship between lorica volume, carbon, nitorgen, and ATP content of tintinnids in Narragansett Bay.* J. Plankton Res., 1984. **6**: p. 859-868.
12. Menden-Deuer, S. and E.J. Lessard, *Carbon to volume relationships for dinoflagellates, diatoms, and other protist plankton.* Limnol. Oceanogr., 2000. **45**(3): p. 569-579.
13. Boraas, M.E., *Population dynamics of food-limited rotifers in two-stage chemostat culture.* Limnol. Oceanogr., 1983. **28**: p. 546–563.
14. Uye, S., N. Nagano, and T. Tamaki, *Geographical and seasonal variations in abundance, biomass and estimated production rates of microzooplankton in the Inland Sea of Japan.* J. Oceanogr. Soc. Jpn., 1996. **52**: p. 689–703.
15. Apprill, A., et al., *Minor revision to V4 region SSU rRNA 806R gene primer greatly increases detection of SAR11 bacterioplankton.* Aquatic Microbial Ecology, 2015. **75**(2): p. 129-137.
16. Parada, A., D.M. Needham, and J.A. Fuhrman, *Every base matters: assessing small subunit rRNA primers for marine microbiomes with mock communities, time-series and global field samples.* Environmental Microbiology, 2016. **18**: p. 1403–1414.
17. Stoeck, T., et al., *Multiple marker parallel tag environmental DNA sequencing reveals a highly complex eukaryotic community in marine anoxic water.* Mol Ecol, 2010. **19 Suppl 1**: p. 21-31.
18. Kozich, J.J., et al., *Development of a dual-index sequencing strategy and curation pipeline for analyzing amplicon sequence data on the MiSeq Illumina sequencing platform.* Applied and Environmental Microbiology 79: 5112–5120, 2013.

19. Lange, V., et al., *Cost-efficient high-throughput HLA typing by MiSeq amplicon sequencing*. BMC Genomics, 2014. **15**(1): p. 63.
20. Bolyen, E., et al., *Reproducible, interactive, scalable and extensible microbiome data science using QIIME 2*. Nature Biotechnology, 2019. **37**(8): p. 852-857.
21. Martin, M., *Cutadapt removes adapter sequences from high-throughput sequencing reads*. EMBnet.journal, 2011. **17**: p. 10-12.
22. Callahan, B.J., et al., *DADA2: High-resolution sample inference from Illumina amplicon data*. Nature Methods, 2016. **13**: p. 581.
23. Pedregosa, F., et al., *Scikit-learn: Machine Learning in Python*. Journal of Machine Learning Research, 2011. **12**: p. 2825-2830.
24. Quast, C., et al., *The SILVA ribosomal RNA gene database project: improved data processing and web-based tools*. Nucleic Acids Res, 2013. **41**: p. D590−D596.
25. Guillou, L., et al., *The Protist Ribosomal Reference database (PR2): a catalog of unicellular eukaryote Small Sub-Unit rRNA sequences with curated taxonomy*. Nucleic Acids Research, 2013. **41**(D1): p. D597-D604.
26. Vaulot, D., et al., *PR2 version 5.0.0*. 2023: Zenodo, 10.5281/zenodo.7805244.
27. Vaulot, D., et al., *metaPR(2) : A database of eukaryotic 18S rRNA metabarcodes with an emphasis on protists*. Mol Ecol Resour, 2022. **22**(8): p. 3188-3201.
28. Faith, D.P., *Conservation evaluation and phylogenetic diversity*. Biological Conservation, 1992. **61**: p. 1–10.
29. Kurtz, Z.D., et al., *Sparse and compositionally robust inference of microbial ecological networks*. PLoS Comput Biol, 2015. **11**(5): p. e1004226.
30. Csardi, G. and T. Nepusz, *The igraph software package for complex network research*. InterJournal, Complex Systems, 2006. **1695**: p. 1-9.
31. Peschel, S., et al., *NetCoMi: network construction and comparison for microbiome data in R*. Brief Bioinform, 2021. **22**(4).
32. Buchan, A., et al., *Master recyclers: features and functions of bacteria associated with phytoplankton blooms*. Nature Reviews Microbiology, 2014. **12**(10): p. 686-698.
33. Teeling, H., et al., *Substrate-Controlled Succession of Marine Bacterioplankton Populations Induced by a Phytoplankton Bloom*. Science, 2012. **336**(6081): p. 608-611.
34. Simpson, A.G.B. and D.J. Patterson, *Ultrastructure and identification of the predatory flagellateColpodella pugnax Cienkowski (Apicomplexa) with a description ofColpodella turpis n. sp. and a review of the genus*. Systematic Parasitology, 1996. **33**(3): p. 187-198.
35. Hess, S., A. Suthaus, and M. Melkonian, *"Candidatus Finniella" (Rickettsiales, Alphaproteobacteria), Novel Endosymbionts of Viridiraptorid Amoeboflagellates (Cercozoa, Rhizaria)*. Appl Environ Microbiol, 2016. **82**(2): p. 659-70.
36. Skovgaard, A., *Dirty tricks in the plankton: Diversity and role of marine parasitic protists*. Acta Protozoologica, 2014. **53**: p. 51-62.
37. Tillmann, U., et al., *Clarifying confusion – Prorocentrum triestinum J.Schiller and Prorocentrum redfieldii Bursa (Prorocentrales, Dinophyceae) are two different species*. European Journal of Phycology, 2021. **57**(2): p. 207-226.
38. Bisanz, J.E., *qiime2R: Importing QIIME2 artifacts and associated data into R sessions*. 2018.
